# Supplementary material for: Rescuing SERCA2 pump deficiency improves bone mechano-responsiveness in type 2 diabetes by shaping osteocyte calcium dynamics
Source: Nat Commun. 2024 Jan 30;15:890. doi: 10.1038/s41467-024-45023-6 (PMC10828510; doi:10.1038/s41467-024-45023-6)
Supplement: Supplementary file 1 — Supplementary information [file 41467_2024_45023_MOESM1_ESM.pdf]

Supplementary Materials for

Rescuing SERCA2 pump deficiency improves bone mechano-responsiveness in type 2 diabetes by shaping osteocyte calcium dynamics

Xi Shao, Yulan Tian, Juan Liu, Zedong Yan, Yuanjun Ding, Xiaoxia Hao, Dan Wang, Liangliang Shen, Erping Luo, X. Edward Guo, Peng Luo, Wenjing Luo, Jing Cai, and Da Jing

*Corresponding author. Email: jingdaasq@126.com

This PDF file includes:

Figs. S1 to S19
Tables. S1 to S3

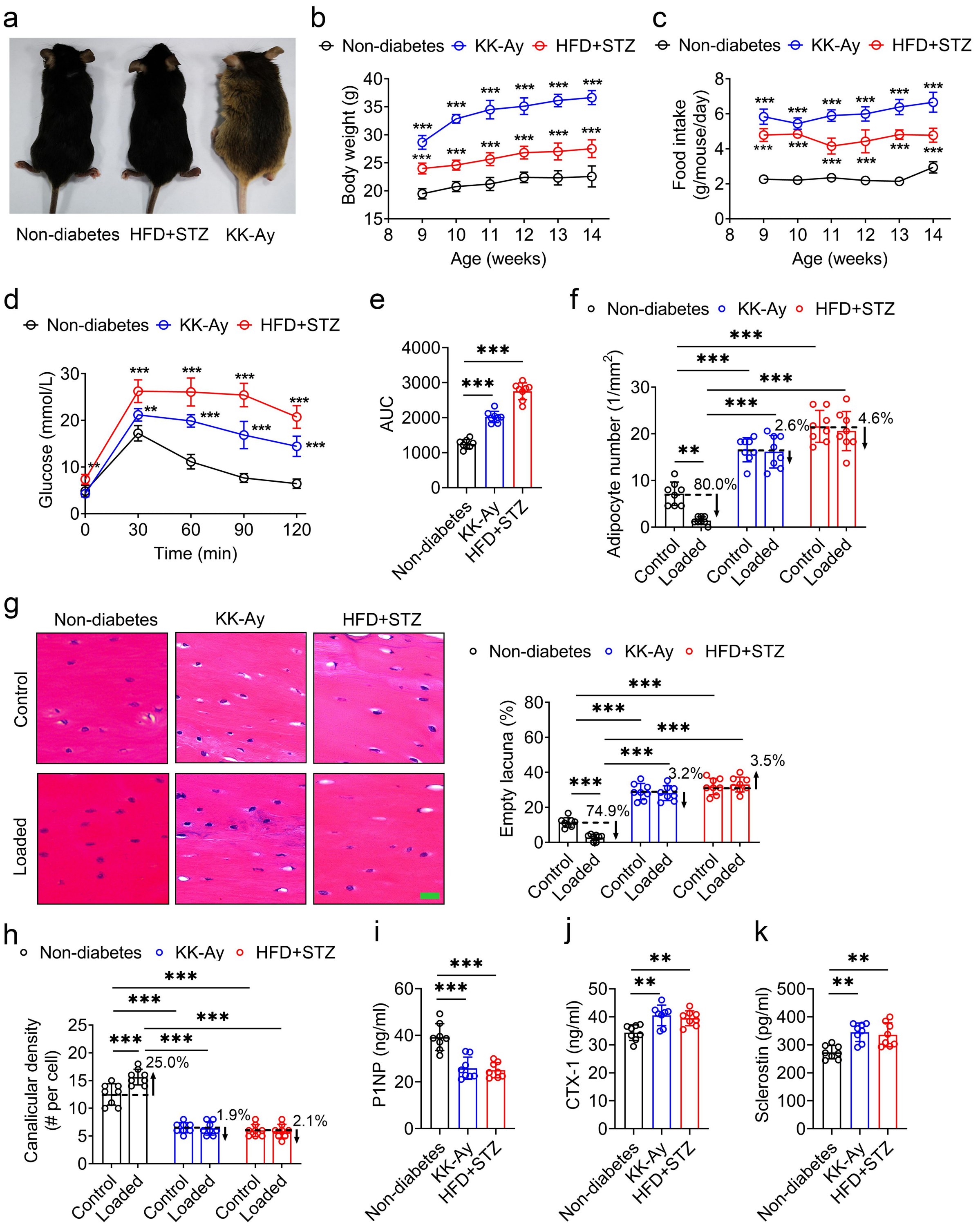

Fig. S1, related to Fig. 1 and Fig. 2. Characterization of the genetically spontaneous and experimentally-induced T2D mice, and effects of exogenous cyclic loading on bone architecture and osteocyte viability inT2D mice. (a) Comparison of the gross appearance of the non-diabetic mice, T2D KK-Ay (genetically spontaneous) mice, and diet/streptozotocin-induced (HFD+STZ, experimentally-induced) T2D mice. **(b and c)** Changes in body weights

and food intake in the non-diabetes, KK-Ay, and HFD+STZ mice. **(d)** Blood glucose levels at 0, 30, 60, 90 and 120 min post the intraperitoneal glucose injection (2 g/kg) to examine glucose tolerance. **(e)** The glucose area under the curve (AUC) calculated based on the glucose tolerance testing. **(f)** Statistical analysis of the number of bone marrow adipocytes in the proximal tibia in diabetic and non-diabetic tibiae following cyclic compressive loading. **(g)** Representative Hematoxylin-eosin (H&E) staining images showing the empty lacuna of osteocytes in diabetic and non-diabetic tibiae following cyclic compressive loading, and the corresponding statistical results. **(h)** Statistical analysis showing the osteocyte canalicular density based on Ploton silver staining in diabetic and non-diabetic tibiae following cyclic compressive loading. **(i-k)** Serum concentrations of P1NP, CTX-1 and sclerostin in the non-diabetes, KK-Ay, and HFD+STZ mice. Graphs represent mean ± SD (n=8 mice per group). **b-e, i-k** **P<0.01, and ***P<0.001 by one-way ANOVA with Bonferroni's post test. **f-h** **P<0.01, and ***P<0.001 by two-way ANOVA with Bonferroni's post test. Specific P values are provided in the Source Data file. Scale bar: **g** 20 μm.

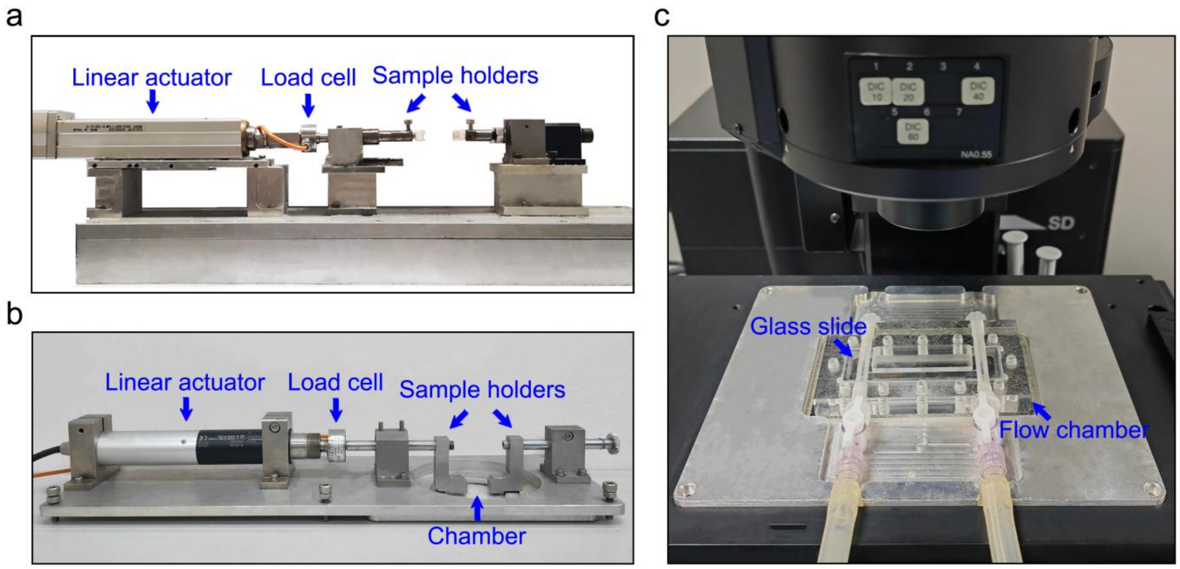

Fig. S2, related to Fig. 1 and Fig. 2. The multi-scale mechanical loading devices used in the current study. (a) The custom-designed cyclic mechanical loading system that generates uniaxial compressive loading on the mouse tibia *in vivo*, which consists of a linear actuator, a linear guide, two sample holders (including an immovable holder and a movable holder), and a loadcell. **(b)** The custom-designed cyclic mechanical loading system that generates uniaxial compressive loading on the intact mouse tibia *ex vivo* for visualizing Ca^{2+} fluorescence in bone cells *in situ*. After incubation with the Ca^{2+} indicator, the *ex vivo* cultured mouse tibia was transferred to the chamber of the mechanical loading device that was filled with the tissue culture medium to maintain the bone cell viability. A novel synchronized loading/imaging technique was used to avoid the drift of confocal objective focus during cyclic loading. **(c)** The laminar fluid flow chamber for visualizing real-time Ca^{2+} dynamics in bone cells *in vitro* in response to fluid shear stress on the cell surface.

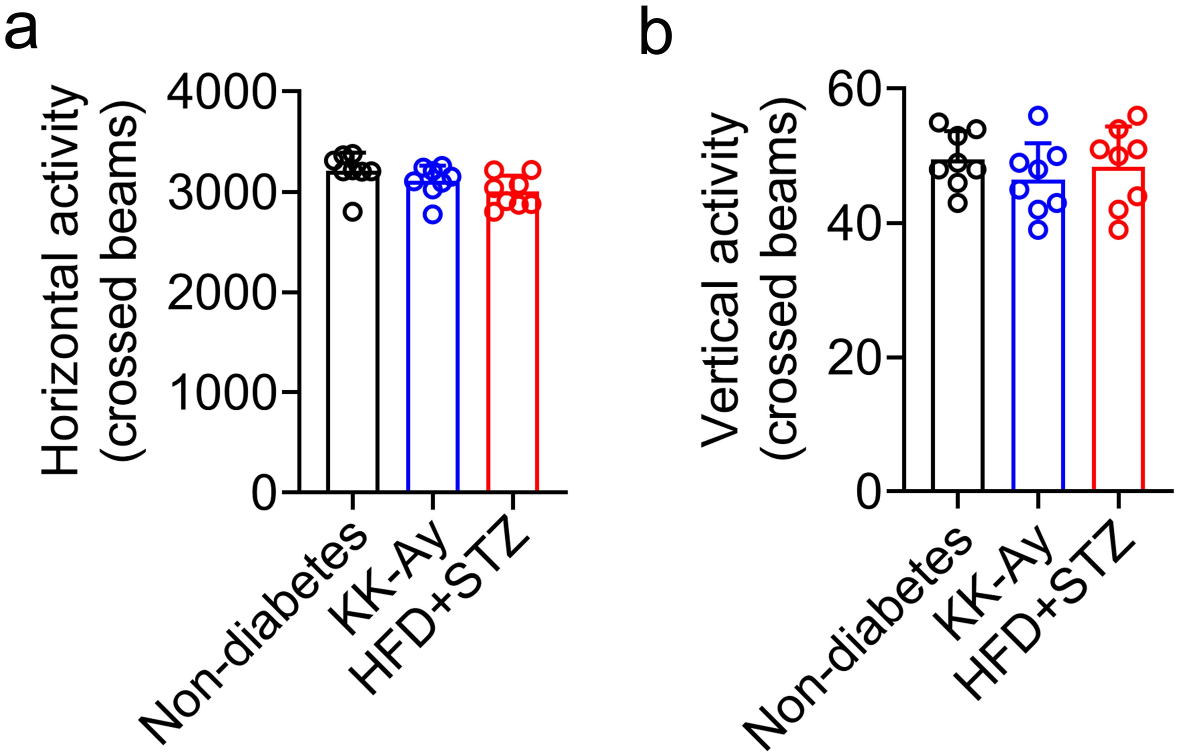

Fig. S3, related to Fig. 1 and Fig. 2. Behavioral open field testing to compare the locomotor activities between normal, genetically spontaneous, and experimentally-induced T2D mice. (a and b) Mice were placed in a Ugo Basile (Model 7420) activity recording system to habituate for 5 min, and then the horizontal activity and the vertical activity were recorded for 60 min by detecting the infrared beam interruptions resulting from the movements of mice. Graphs represent mean ± SD (*n*=8 mice per group). Statistical analysis was performed using one-way ANOVA with Bonferroni's post test.

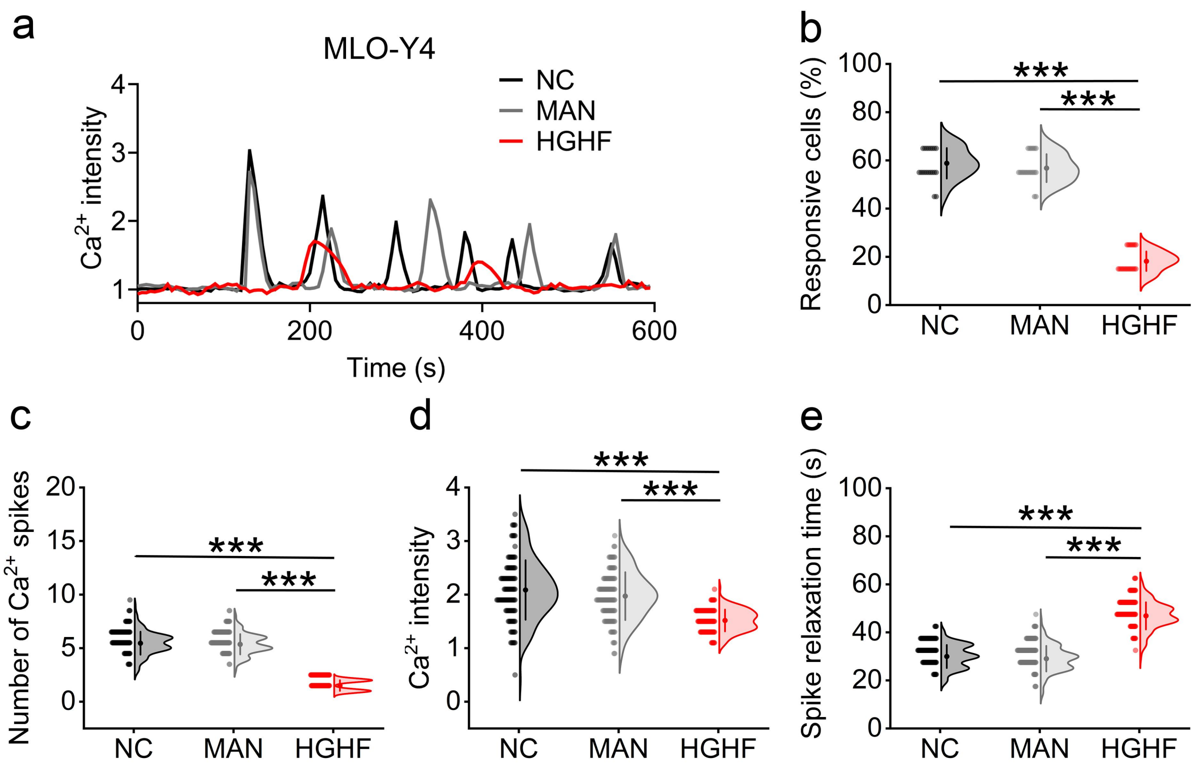

Fig. S4, related to Fig. 3. The intracellular Ca^{2+} oscillatory dynamics in osteocytic MLO-Y4 cells treated with high glucose and high fat under oscillatory fluid flow stimulation. (a) Representative real-time intracellular Ca^{2+} signaling curves of high glucose and high fat (HGHF)-treated osteocytes subjected to oscillatory fluid flow stimulation at 2 Pa and 1 Hz. **(b-e)** The corresponding quantitative data of intracellular Ca^{2+} oscillatory dynamics, including the percentage of responsive cells, the number of Ca^{2+} spikes, average Ca^{2+} intensity, and the relaxation time of Ca^{2+} spikes. Graphs represent mean ± SD (*n*=120 cells per group). ****P*<0.001 by one-way ANOVA with Bonferroni' s post test. Specific *P* values are provided in the Source Data file.

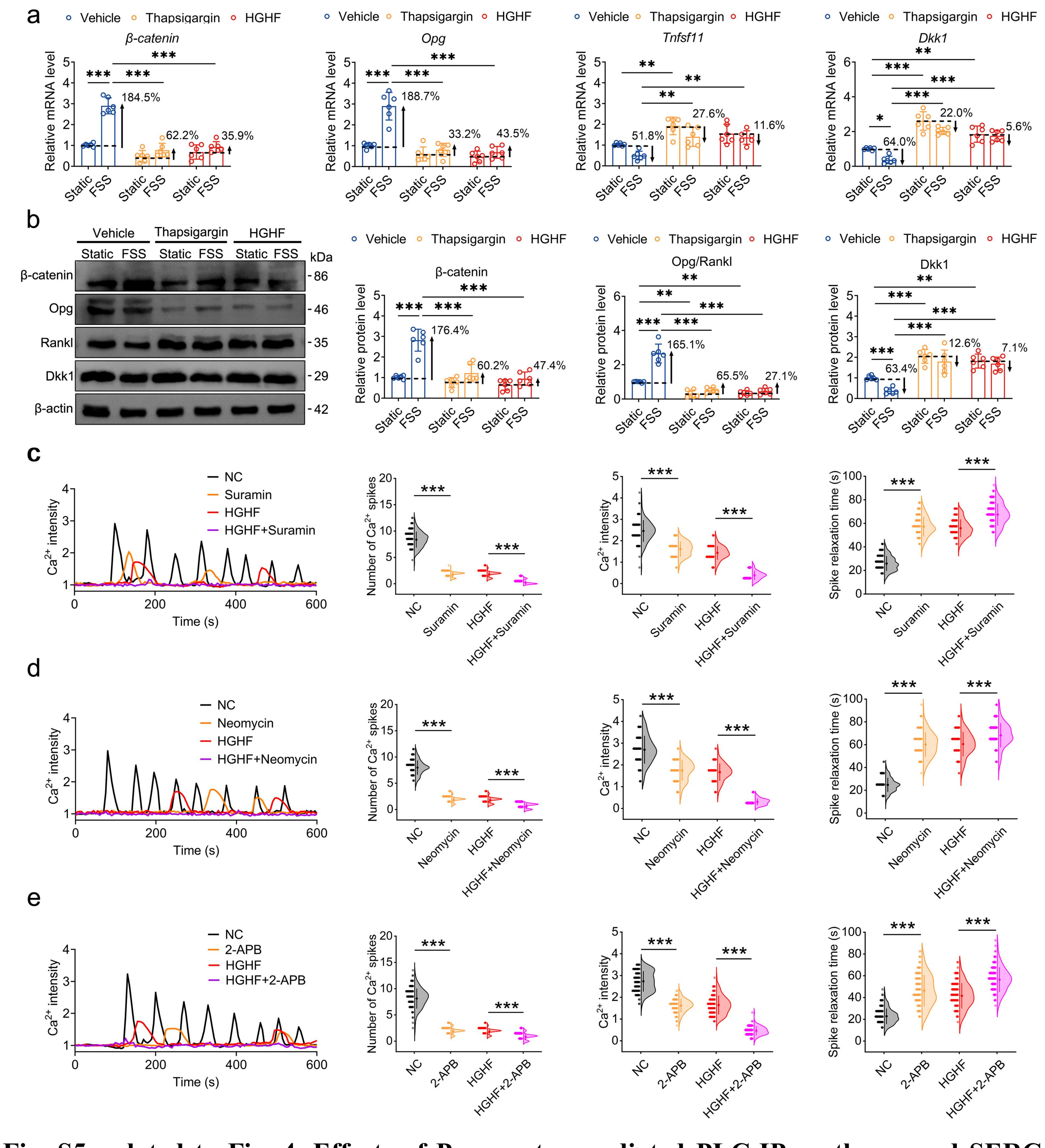

Fig. S5, related to Fig. 4. Effects of P_2 receptor-mediated PLC-IP_3 pathway and SERCA Ca^{2+} pumps in the endoplasmic reticulum on mechanoresponse in normal and high glucose and high fat (HGHF)-treated osteocytes. (a and b) The gene and protein expression in MLO-Y4 osteocytic cells treated with HGHF and thapsigargin (the SERCA antagonist) in response to fluid shear stress (2 Pa) via qRT-PCR and western blotting assays, including β-catenin, OPG, RANKL, and DKK1. **(c-e)** Intracellular Ca^{2+} oscillatory dynamics in normal and HGHF-treated MLO-Y4 cells incubated with suramin (a non-selective P_2R antagonist), neomycin (a PLC inhibitor), and 2-aminoethoxydiphenyl borate (2-APB, an IP_3R antagonist) under fluid flow stimulation, respectively. Graphs represent mean ± SD (**a**, **b**: *n*=6 biologically independent

replicates; **c-e**: n=120 cells per group). **a, b** *$P<0.05$, **$P<0.01$ and ***$P<0.001$ by two-way ANOVA with Bonferroni's post test. **c-e** ***$P<0.001$ by one-way ANOVA with Bonferroni's post test. Specific *P* values are provided in the Source Data file.

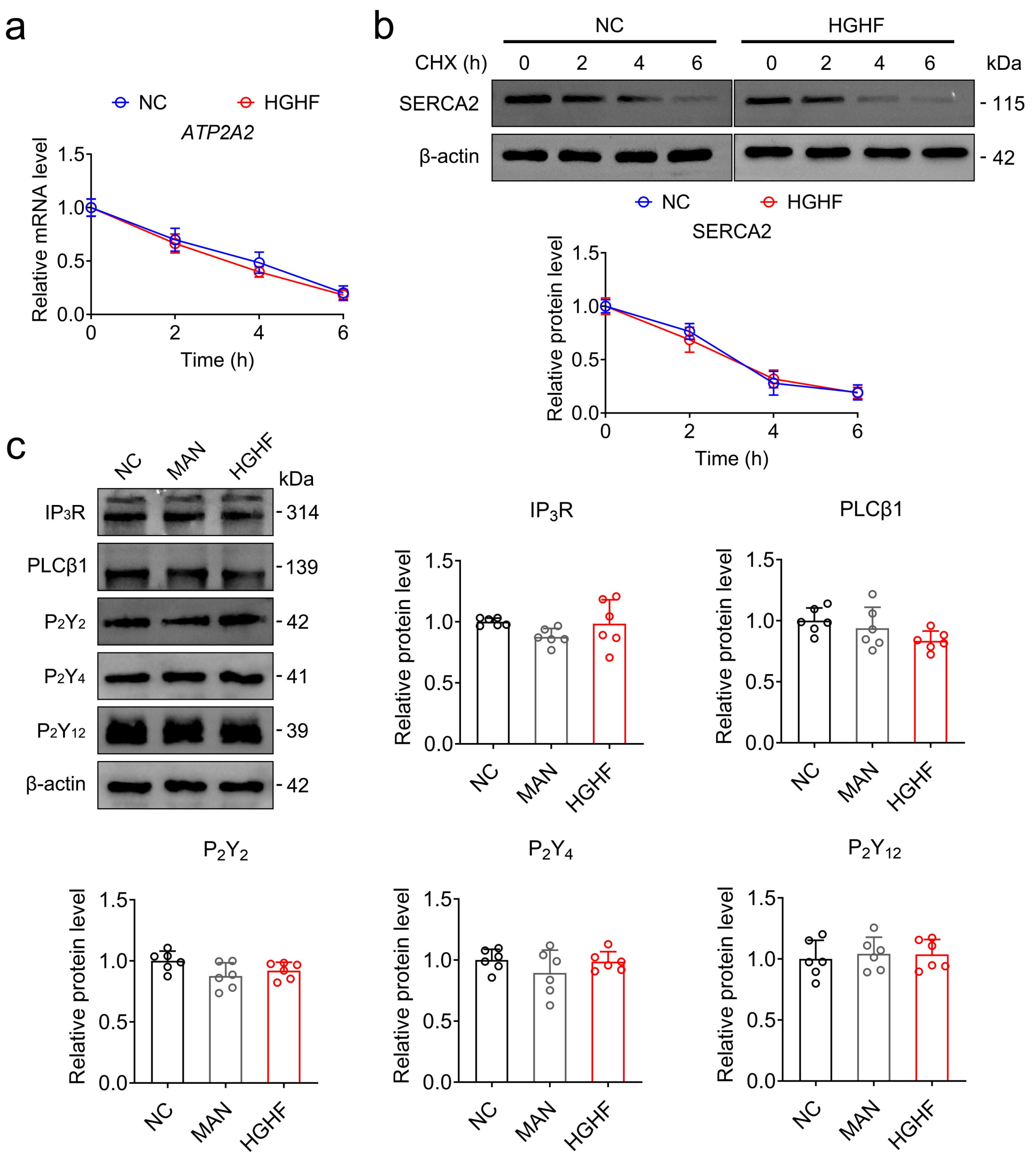

Fig. S6, related to Fig. 4. Effects of high glucose and high fat (HGHF) on the gene and protein stability of SERCA2 and the expression of key proteins in the P_2Y-PLC-IP_3 pathway in osteocytes. (a) The *ATP2A2* mRNA stability analysis in MLO-Y4 cells at 0, 2, 4, and 6 h after actinomycin D treatment under the normal or HGHF condition. **(b)** The protein stability analysis of SERCA2 in MLO-Y4 cells at 0, 2, 4, and 6 h after cycloheximide treatment under the normal or HGHF condition. (n=6 biologically independent replicates). **(c)** The protein expression of P_2Y isoforms (P_2Y_2, P_2Y_4, and P_2Y_{12}, three major functional isoforms expressed in osteocytes), PLCβ1 (P_2Y-downstream effector isoform), and IP_3R in normal, mannitol-treated, and HGHF-treated MLO-Y4 cells via western blotting assays. Graphs represent mean ± SD (n=6 biologically independent replicates). **a, b** Statistical analysis was performed using Student's t test. **c** Statistical analysis was performed using one-way ANOVA with Bonferroni's post test.

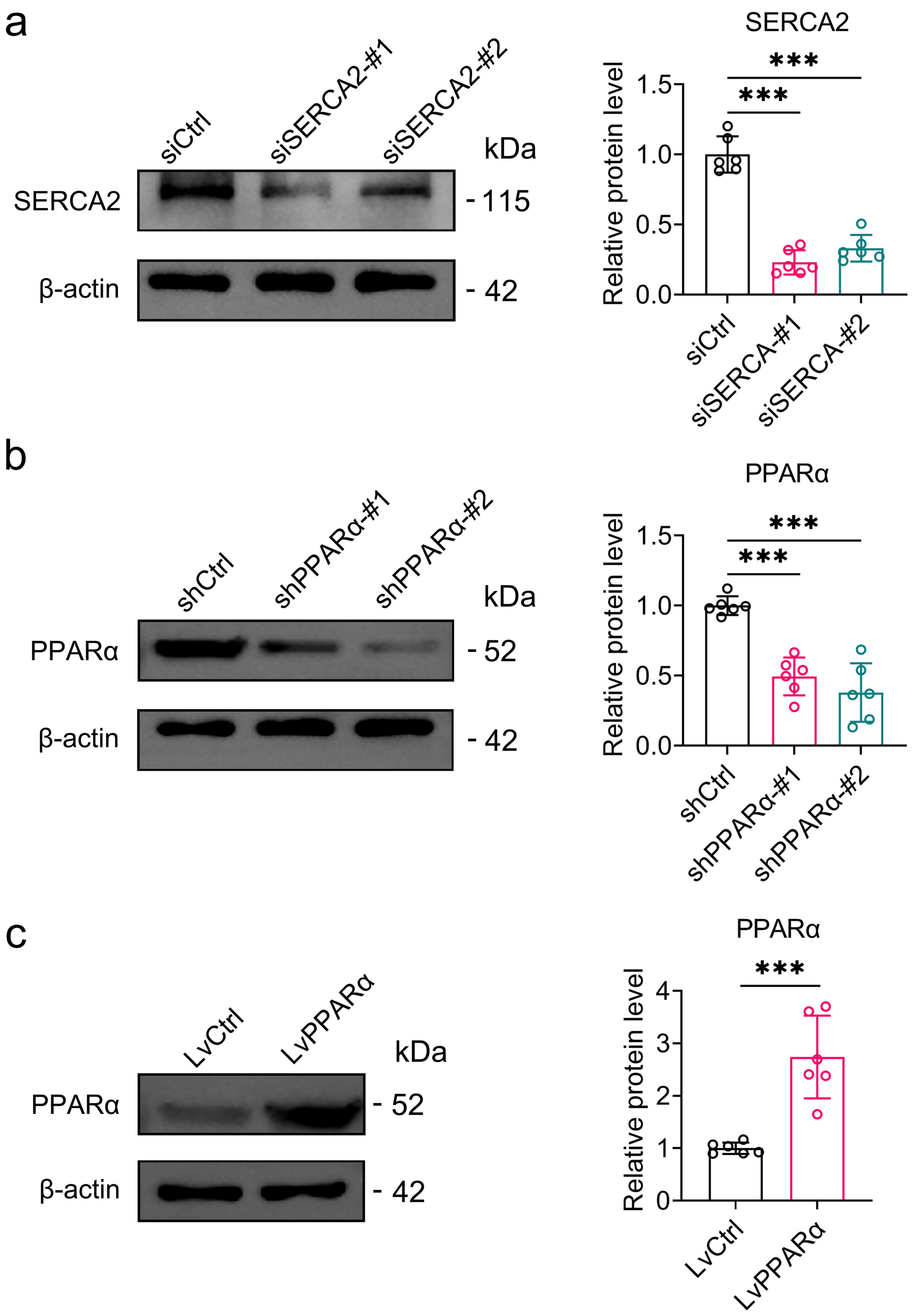

Fig. S7, related to Fig. 4 and Fig. 9. The gene overexpression and knockdown efficiency for the *in vitro* experiments in MLO-Y4 osteocytic cells. (a) Western blotting for examining the knockdown efficiency of *ATP2A2* following siRNA transfection. **(b)** Western blotting for testing the knockdown efficiency of *PPARα* following the lentivirus transfection. **(c)** Western blotting for testing the overexpression efficiency of *PPARα* following the lentivirus transfection. Graphs represent mean ± SD (n=6 biologically independent replicates). **a, b** ***P<0.001 by one-way ANOVA with Bonferroni's post test. **c** ***P<0.001 by two-tailed Student's t test. Specific P values are provided in the Source Data file.

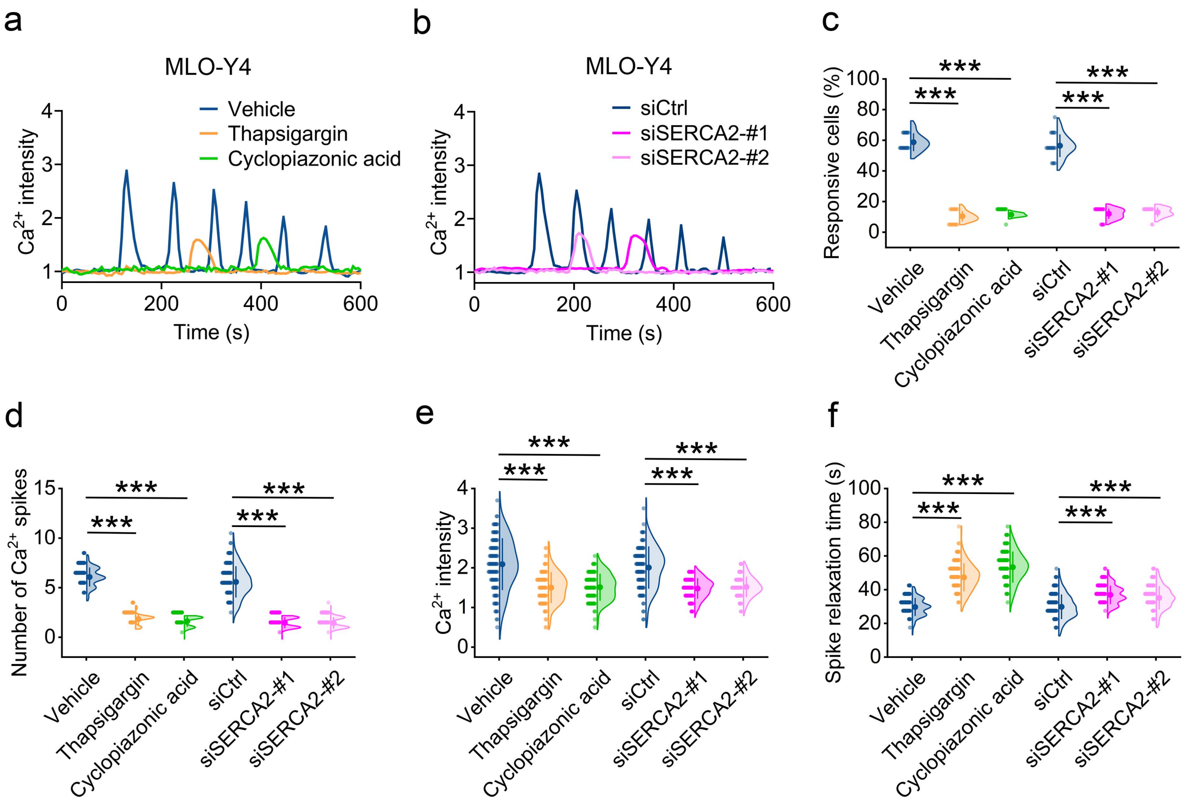

Fig. S8, related to Fig. 4. Effects of SERCA2 on the mediation of intracellular Ca^{2+} dynamics in osteocytic MLO-Y4 cells in response to oscillatory fluid flow stimulation. (a) Representative intracellular Ca^{2+} signaling curves in osteocytes *in vitro* incubated with two specific inhibitors of SERCA to deplete intracellular Ca^{2+} stores (i.e., thapsigargin and cyclopiazonic acid) or DMSO (vehicle control) under oscillatory fluid flow stimulation (2 Pa and 1 Hz). **(b)** Representative intracellular Ca^{2+} signaling curves in osteocytes *in vitro* infected with siSERCA2 or siCtrl under oscillatory fluid flow stimulation. **(c-f)** The corresponding quantitative data of intracellular Ca^{2+} oscillatory dynamics, including the percentage of responsive cells, the number of Ca^{2+} spikes, average Ca^{2+} intensity, and the relaxation time of Ca^{2+} spikes. Graphs represent mean ± SD (n=120 cells per group). ***P<0.001 by one-way ANOVA with Bonferroni' s post test. Specific P values are provided in the Source Data file.

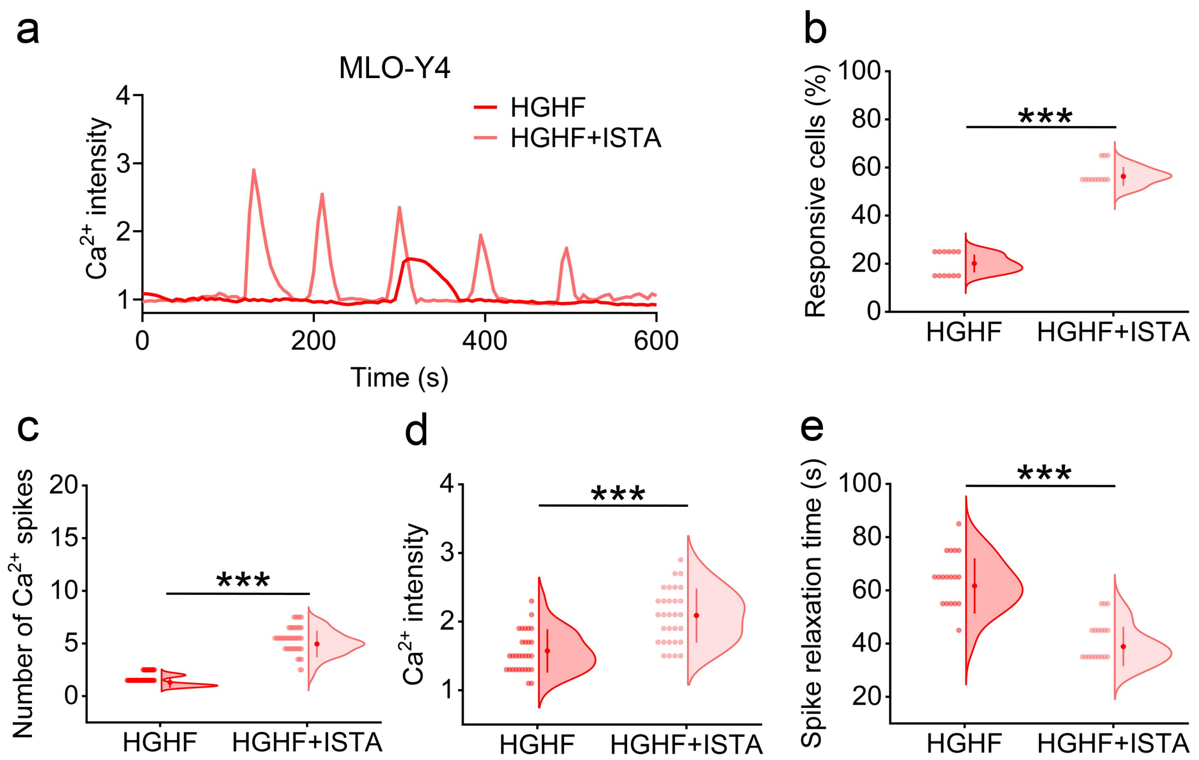

Fig. S9, related to Fig. 5. Effects of the SERCA2 agonist ISTA on intracellular Ca^{2+} dynamics in osteocytic MLO-Y4 cells treated with high glucose and high fat in response to oscillatory fluid flow stimulation. (a) Representative intracellular Ca^{2+} signaling curves in high glucose and high fat (HGHF)-treated osteocytes supplemented with the SERCA2 agonist ISTA (1 μM) under oscillatory fluid flow stimulation (2 Pa and 1 Hz). **(b-e)** The corresponding quantitative data of intracellular Ca^{2+} oscillatory dynamics, including the percentage of responsive cells, the number of Ca^{2+} spikes, average Ca^{2+} intensity, and the relaxation time of Ca^{2+} spikes. Graphs represent mean ± SD (*n*=120 cells per group). ****P*<0.001 by Student's t test. Specific *P* values are provided in the Source Data file.

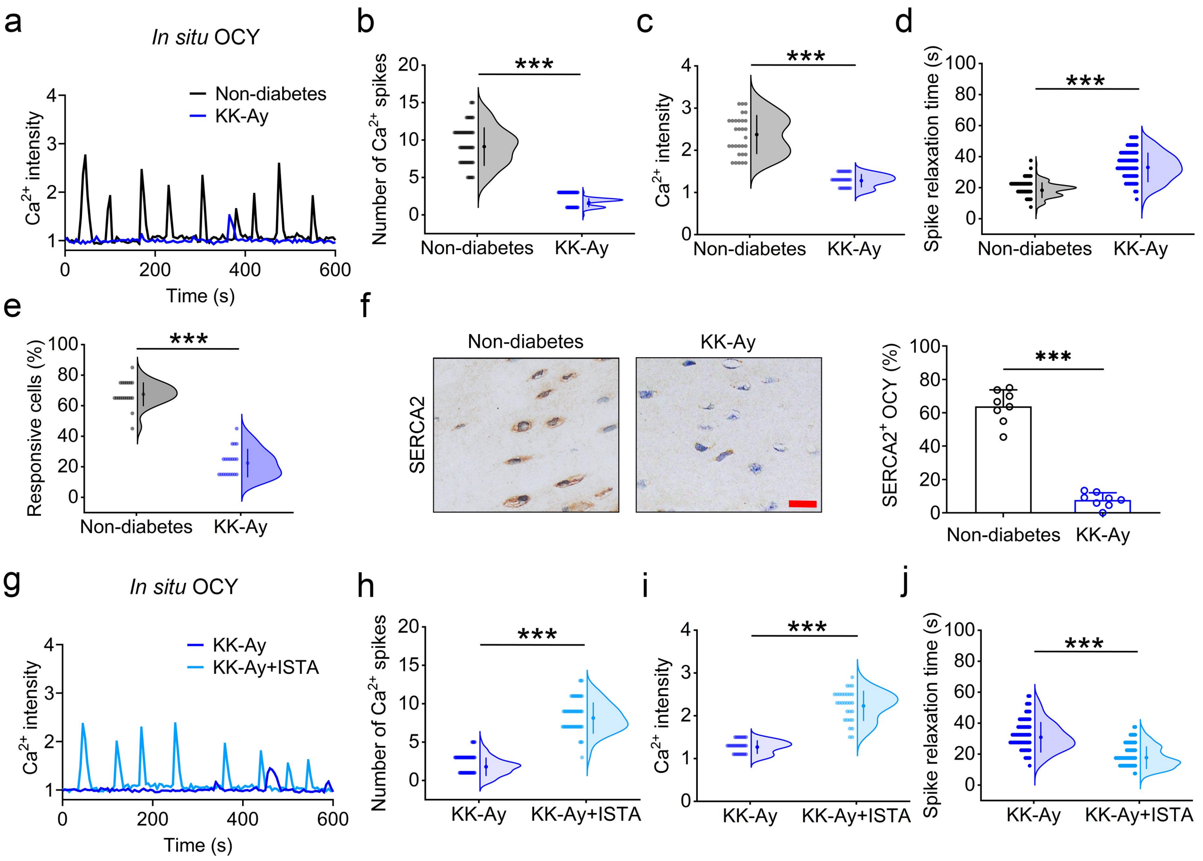

Figure S10, related to Fig. 3, Fig. 4, and Fig. 5. The osteocyte mechano-responsiveness and SERCA2 expression in female KK-Ay mice, and effects of the SERCA2 agonist ISTA on osteocyte Ca^{2+} oscillatory responses to mechanical loading in female KK-Ay mice. (a-e) Representative intracellular Ca^{2+} signaling curves of osteocytes *in situ* in the tibiae of female non-diabetic and KK-Ay mice in response to cyclic mechanical loading, and the corresponding quantitative data of intracellular Ca^{2+} oscillatory dynamics. **(f)** Immunohistochemical staining for the SERCA2 expression in osteocytes in the tibiae of female non-diabetic, KK-Ay, and HFD+STZ mice. **(g-j)** Representative intracellular Ca^{2+} signaling curves of osteocytes *in situ* in the tibiae of female KK-Ay mice treated with the SERCA2 agonist istaroxime (ISTA, 1 μM) in response to axial cyclic compressive loading. Graphs represent mean ± SD (**b-e, h-j**: n=120 cells per group; **f:** n=8 mice per group). **b-f, h-j** ***P<0.001 by Student's t test. Specific P values are provided in the Source Data file. Scale bars: **f** 20 μm.

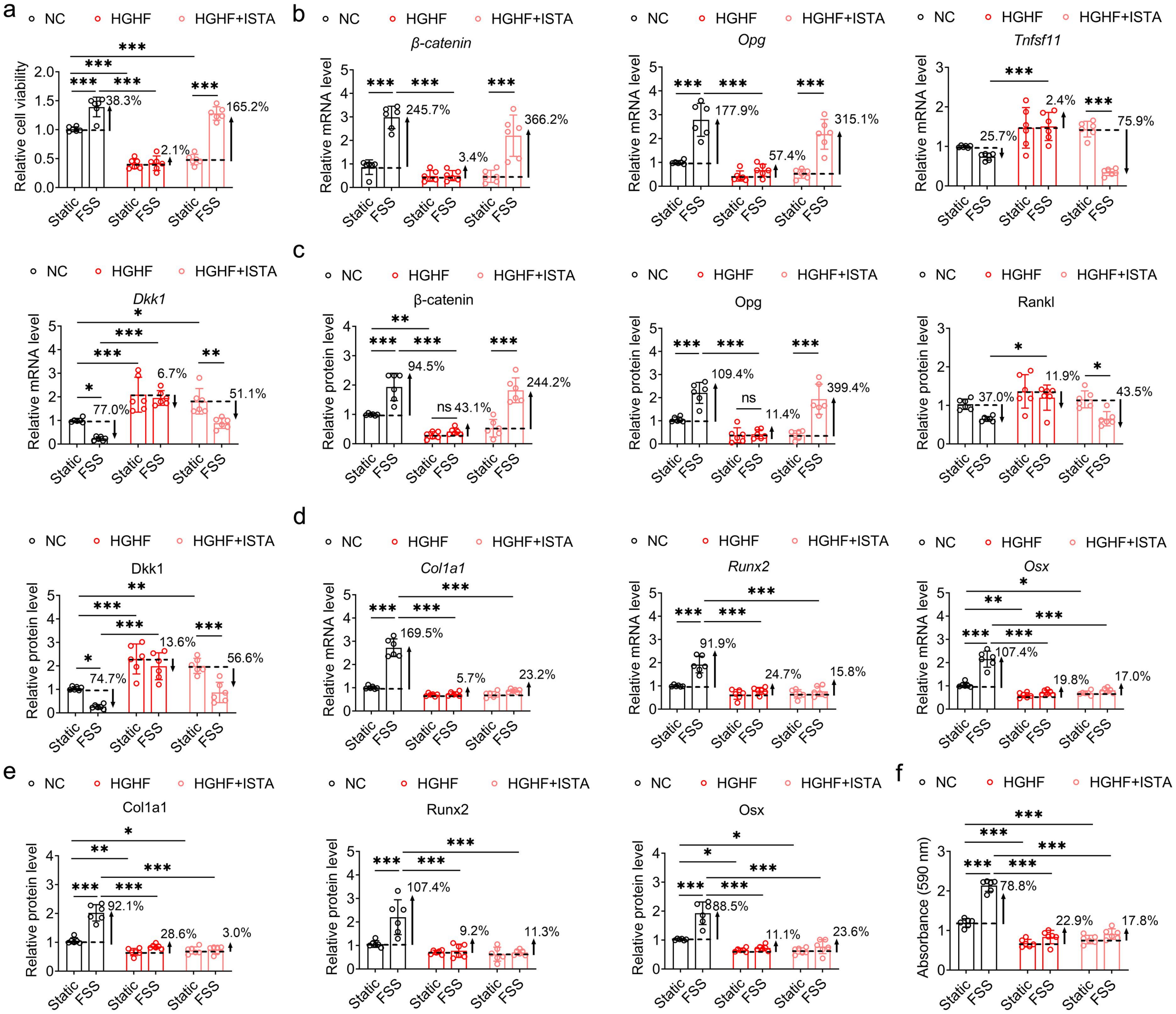

Fig. S11, related to Fig. 5. Effects of the SERCA2 agonist ISTA on the biological activities in high glucose and high fat (HGHF)-exposed osteocytes and osteoblasts in response to fluid shear stress *in vitro*. (a) The CCK-8-based viability assays in MLO-Y4 osteocytic cells treated with HGHF and the SERCA2 agonist ISTA (1 μM) in response to fluid shear stress stimulation (2 Pa). **(b and c)** Statistical analyses of the gene and protein expression in MLO-Y4 osteocytic cells treated with HGHF and ISTA in response to fluid shear stress via qRT-PCR and western blotting assays, including β-catenin, OPG, RANKL, and DKK1. **(d and e)** Statistical analyses of the gene and protein expression in primary osteoblasts treated with HGHF and ISTA in response to fluid shear stress, including Col1a1, Osx, and Runx2. **(f)** Statistical analyses of the absorbance of Alizarin red staining in primary osteoblasts treated with HGHF and ISTA in response to fluid shear stress. Graphs represent mean ± SD (*n*=6 biologically independent replicates). **P*<0.05, ***P*<0.01, and ****P*<0.001 by two-way ANOVA with Bonferroni's post test. Specific *P* values are provided in the Source Data file.

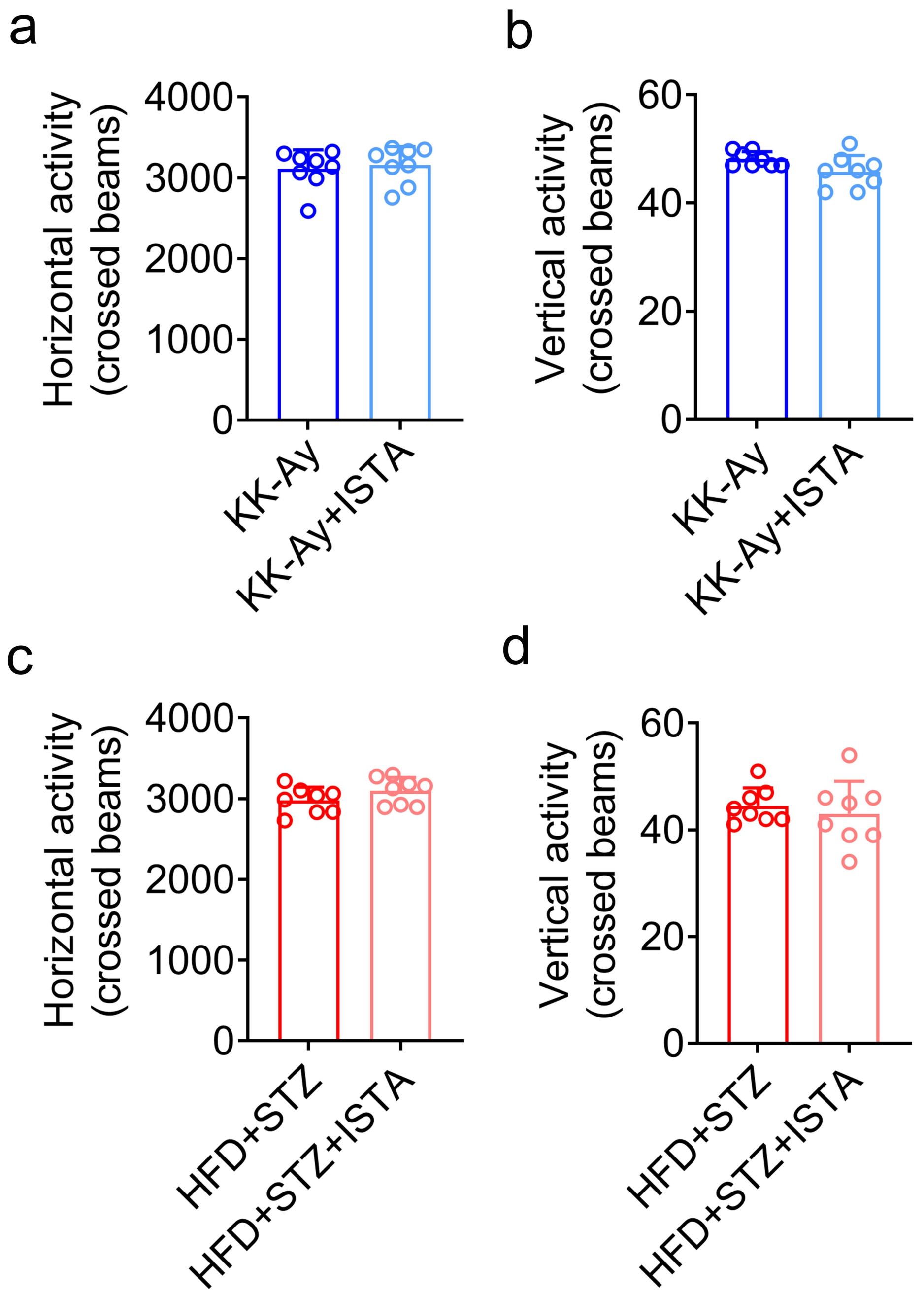

Fig. S12, related to Fig. 6 and Fig. 7. Effects on ISTA treatment on locomotor activities in genetically spontaneous and experimentally-induced T2D mice via behavioral open field testing. (a and b) KK-Ay mice treated with the SERCA2 agonist ISTA were placed in a Ugo Basile (Model 7420) activity recording system to habituate for 5 min, and then the horizontal activity and the vertical activity of mice were recorded for 60 min. **(c and d)** The horizontal activity and the vertical activity of high-fat diet/streptozotocin-treated mice infused with the SERCA2 agonist ISTA were detected. Graphs represent mean ± SD (*n*=8 mice per group). Statistical analysis was performed using Student's t test.

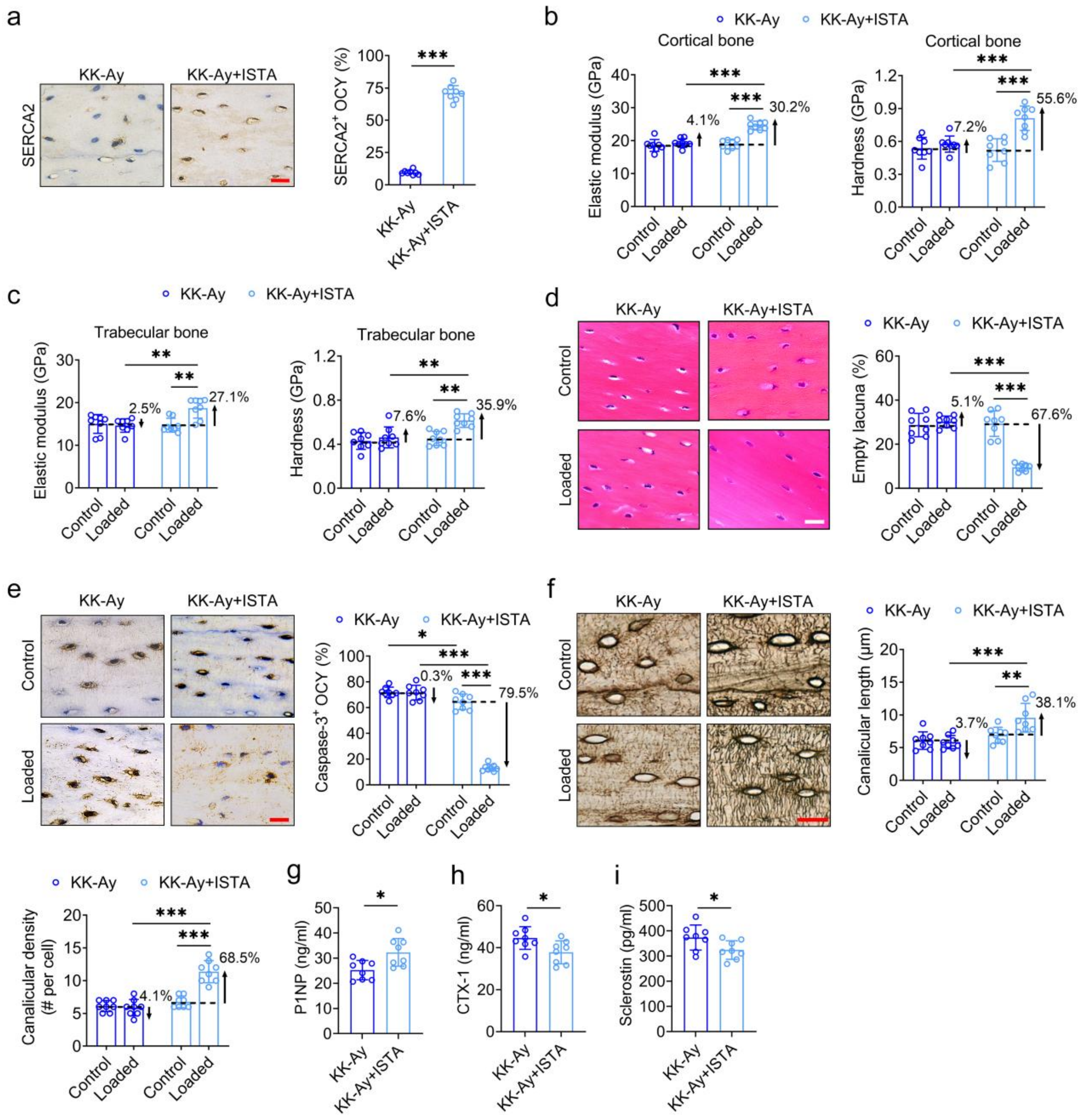

Fig. S13, related to Fig. 6. Effects of ISTA treatment on the mechanical properties, bone turnover and the number and activities of osteocytes in the tibiae of T2D KK-Ay mice in response to exogenous cyclic loading. (a) Immunohistochemical staining for the SERCA2 expression in osteocytes in the tibiae of KK-Ay mice treated with the SERCA2 agonist ISTA. **(b and c)** Nanoindentation testing for local tissue-level material properties in trabecular bone and cortical bone. **(d)** H&E staining for showing the empty lacuna in tibial osteocytes. **(e)** Immunohistochemical staining showing the protein expression of Caspase-3 in osteocytes in tibial cortical bone matrix. **(f)** Ploton silver staining for showing the morphology of tibial osteocyte canalicular network. **(g-i)** ELISA assays for quantifying serum concentrations of P1NP, CTX-1 and sclerostin. Graphs represent mean ± SD (n=8 mice per group). **a, g-i** *P<0.05 and ***P<0.001 by two-tailed Student's t test. **b-f** *P<0.05, **P<0.01 and ***P<0.001 by two-way ANOVA with Bonferroni's post test. Specific P values are provided in the Source Data file. Scale bars: **a, d, e, f** 20 μm.

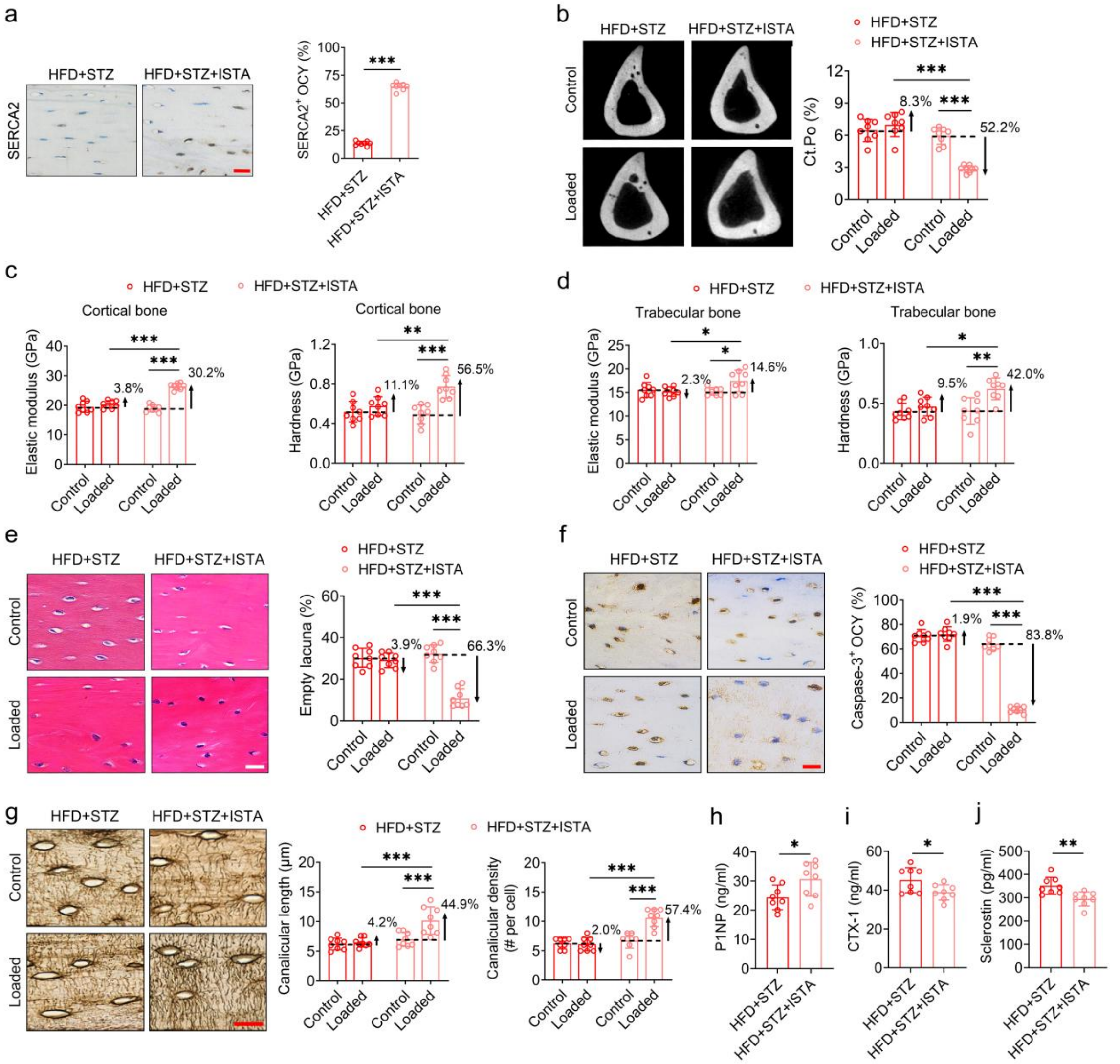

Fig. S14, related to Fig. 7. Effects of ISTA treatment on bone architecture, mechanical properties, bone turnover and the number and activities of osteocytes in high-fat diet/streptozotocin-induced T2D mice in response to exogenous cyclic loading. (a) Immunohistochemical staining for the SERCA2 expression in osteocytes in the tibiae of high-fat diet/streptozotocin-treated mice infused with the SERCA2 agonist ISTA. **(b)** Micro-CT scanning for tibial cortical bone porosity. **(c and d)** Nanoindentation testing for local tissue-level material properties in tibial trabecular bone and cortical bone. **(e)** H&E staining for showing the empty lacuna within tibial cortical bone matrix. **(f)** Immunohistochemical staining for the Caspase-3 expression of osteocytes within tibial cortical bone matrix. **(g)** Ploton silver staining for showing the morphology of tibial osteocyte canalicular network. **(h-j)** ELISA assays for quantifying serum concentrations of P1NP, CTX-1 and sclerostin. Graphs represent mean ± SD (*n*=8 mice per group). **a, h-j** **P*<0.05 ***P*<0.01, and ****P*<0.001 by two-tailed Student's t test. **b-g** **P*<0.05, ***P*<0.01 and ****P*<0.001 by two-way ANOVA with Bonferroni's post test. Specific *P* values are provided in the Source Data file. Scale bars: **a, e, f, g** 20 μm.

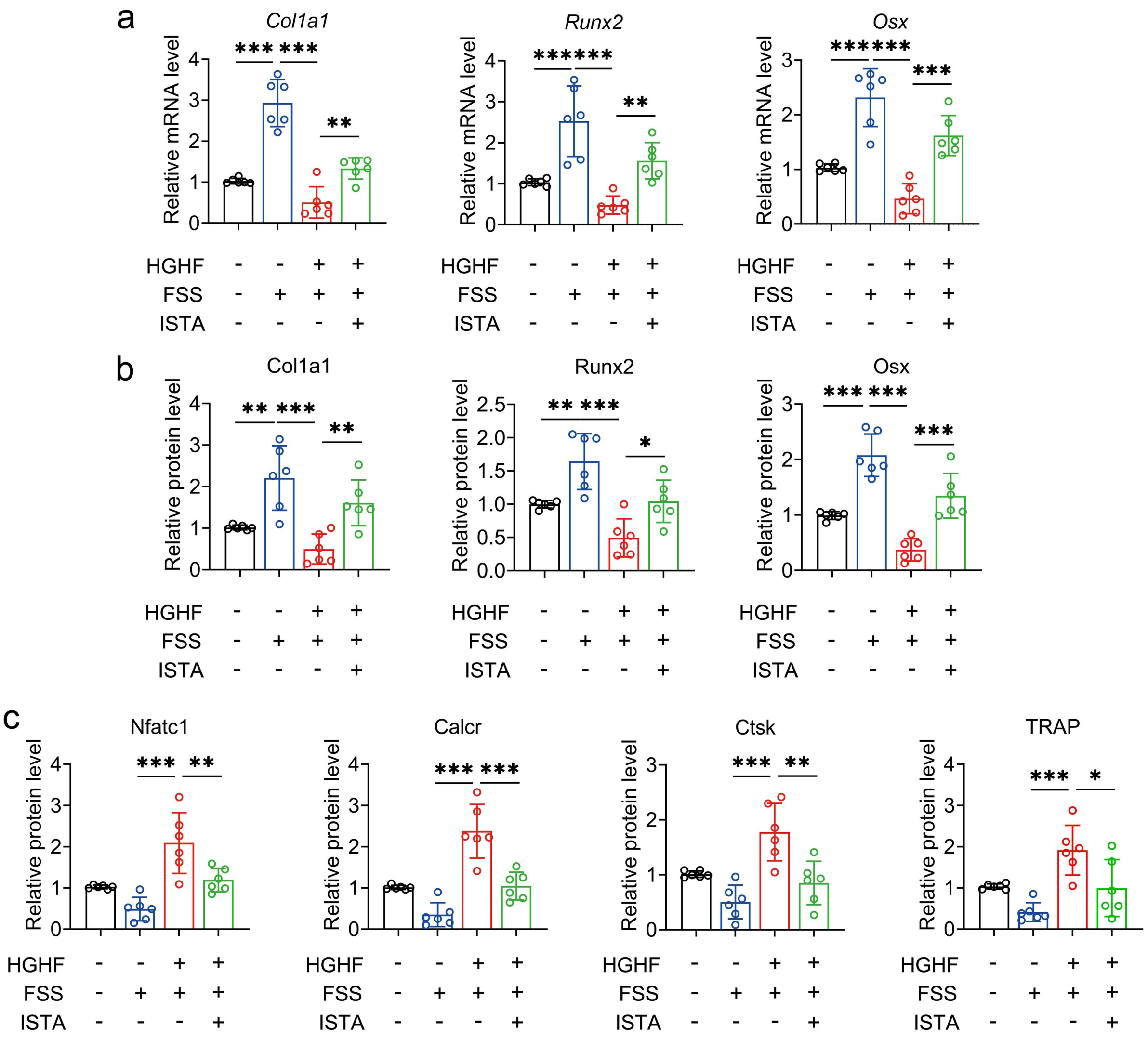

Fig. S15, related to Fig. 7. Effects of the conditioned medium collected from high glucose and high fat (HGHF)-exposed MLO-Y4 osteocytic cells treated with ISTA in response to fluid flow stimulation on the expression of key cytokines of HGHF-exposed primary osteoblasts and RAW264.7 cells. (a and b) The gene and protein expression of osteogenic differentiation-related markers in HGHF-exposed primary osteoblasts, including Col1a1, Osx, and Runx2. **(c)** The protein expression of osteoclastogenesis-related markers, including cathepsin K, TRAP, NFATc1, and calcitonin receptor in HGHF-exposed RAW264.7 cells. Graphs represent mean ± SD (*n*=6 biologically independent replicates). **P*<0.05, ***P*<0.01, and ****P*<0.001 by one-way ANOVA with Bonferroni's post test. Specific *P* values are provided in the Source Data file.

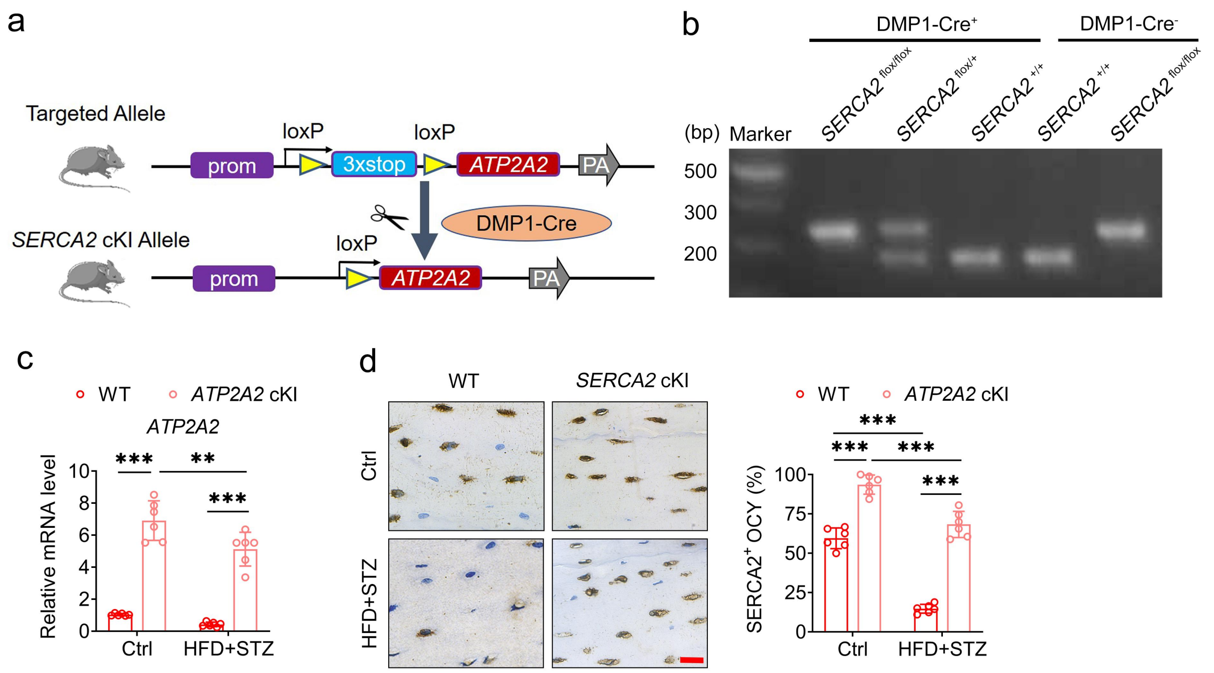

Fig. S16, related to Fig. 8. Generation and confirmation of mice with osteocytes-specific overexpression of SERCA2. (a) Schematic of the overexpression of SERCA2 by recombining with DMP1-Cre mice. The SERCA2 in the mutants with the floxed allele were specifically overexpressed in osteocytes when mated with the mice carrying DMP1-Cre recombinase gene. **(b)** Genotyping the offspring after mating transgenic Cre and loxp mice by agarose gel electrophoresis assays. **(c and d)** The confirmation of osteocytes-specific increase in the gene and protein expression of SERCA2 in tibiae sections via qRT-PCR and immunofluorescence staining assays. SERCA2$^{flox/flox}$; DMP1-Cre^{-}: wild type (WT) mice. SERCA2$^{flox/flox}$; DMP1-Cre^{+}: *SERCA2* cKI mice. Graphs represent mean ± SD (*n*=6 mice per group). ***P*<0.01 and ****P*<0.001 by two-way ANOVA with Bonferroni's post test. Specific *P* values are provided in the Source Data file. Scale bar: **d** 20 μm.

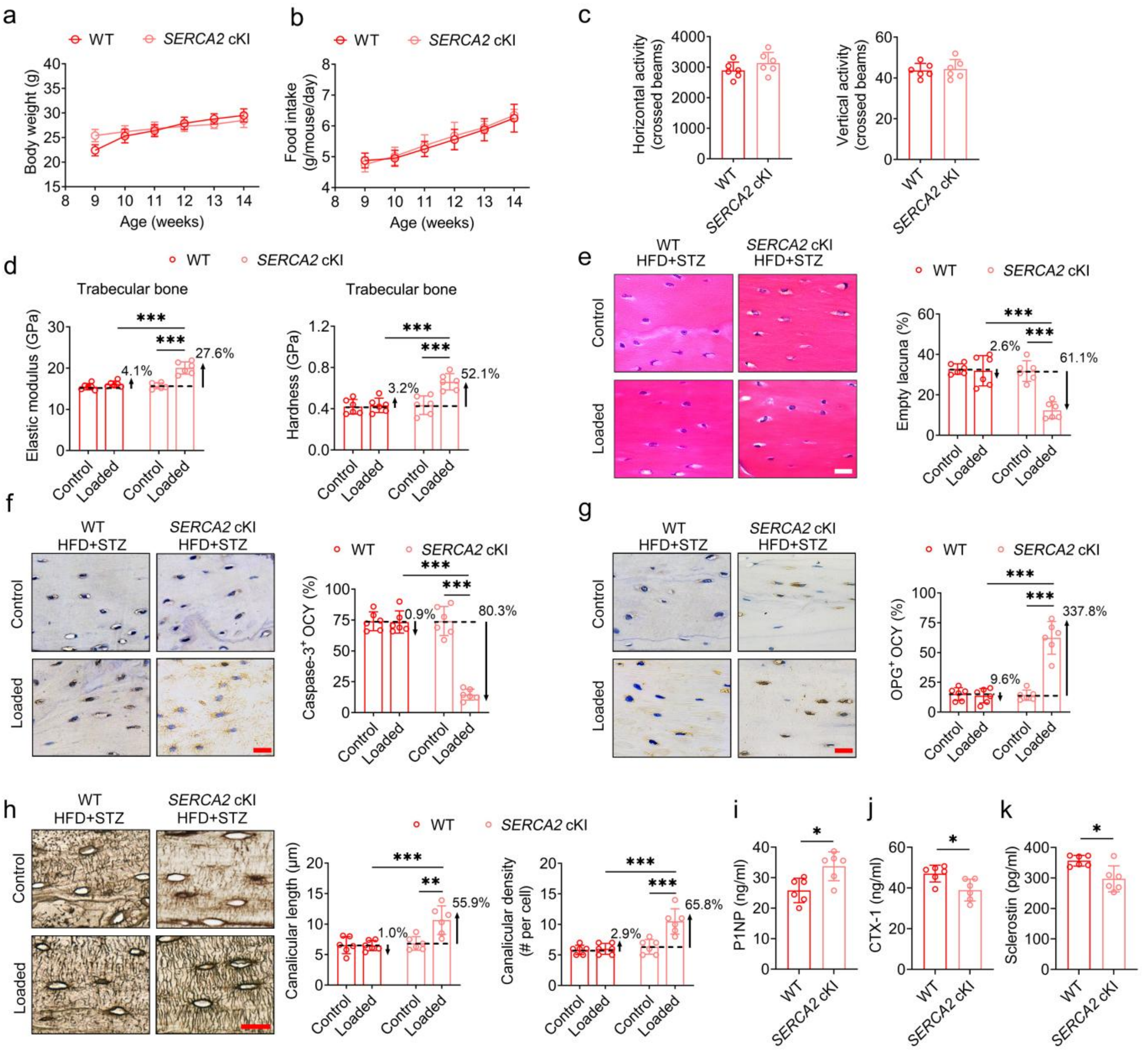

Fig. S17, related to Fig. 8. Comparison of biological behaviors and bone phenotype between the mice with SERCA2 overexpression in osteocytes (SERCA2 cKI) and the corresponding wild type mice (WT) with high-fat diet/streptozotocin treatment. (a-c) The body weight, food intake, and locomotor activities in SERCA2 cKI and WT mice with high-fat diet/streptozotocin treatment. **(d)** Nanoindentation testing for local tissue-level material properties in trabecular bone. **(e)** H&E staining for showing the empty lacuna in tibial osteocytes. **(f and g)** Immunohistochemical staining for the Caspase-3 and OPG expression in tibial osteocytes. **(h)** Ploton silver staining for showing tibial osteocyte canalicular network. **(i-k)** ELISA assays for quantifying serum concentrations of P1NP, CTX-1 and sclerostin. Graphs represent mean ± SD (*n*=6 mice per group). **a-c, i-k** **P*<0.05 by two-tailed Student's t test. **d-h** ***P*<0.01 and ****P*<0.001 by two-way ANOVA with Bonferroni's post test. Specific *P* values are provided in the Source Data file. Scale bars: **e, f, g, h** 20 μm.

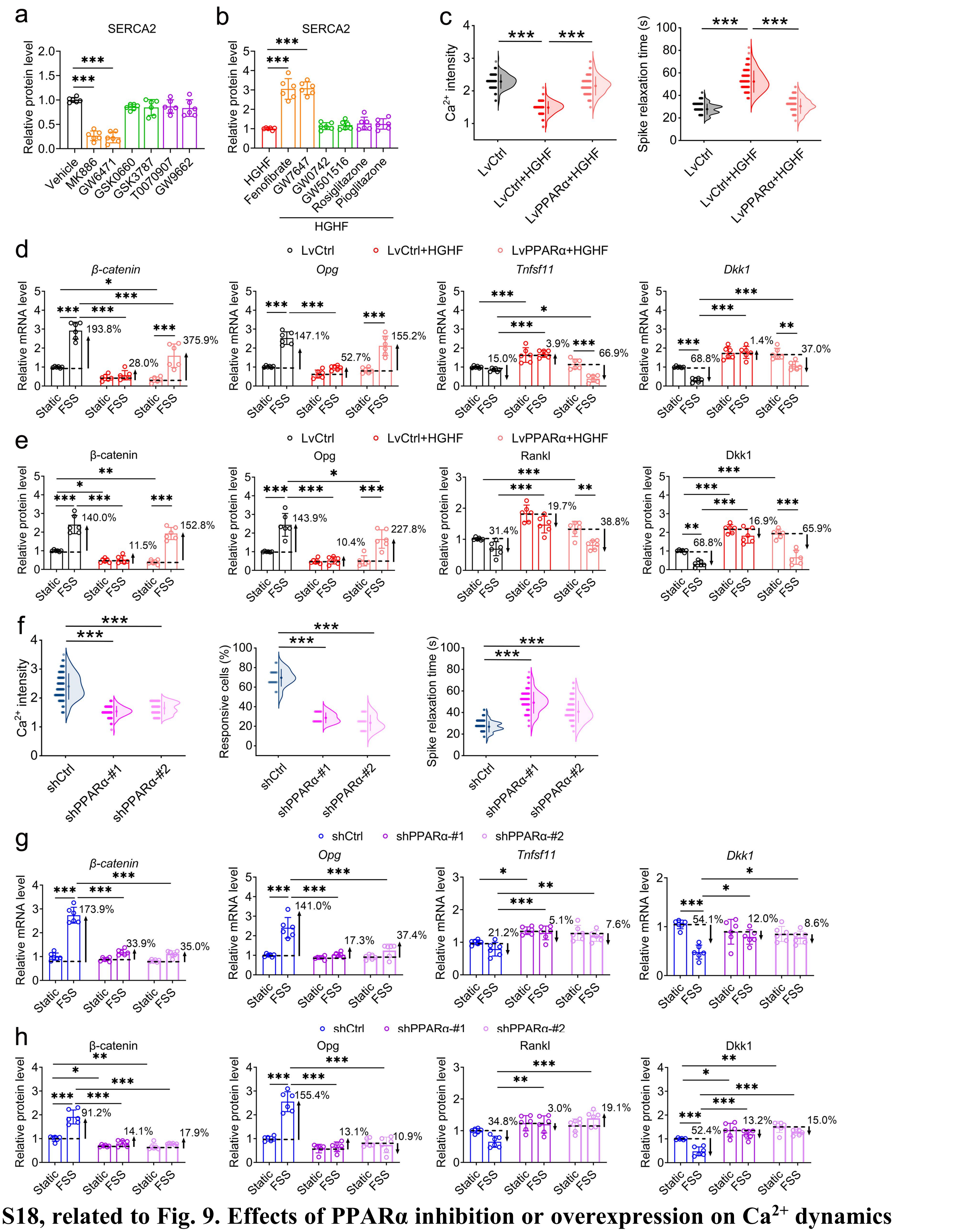

Fig. S18, related to Fig. 9. Effects of PPARα inhibition or overexpression on Ca^{2+} dynamics and the expression of SERCA2 and osteocyte-related cytokines in normal or HGHF-exposed MLO-Y4 cells. (a) Western blotting statistical analyses of the SERCA2 expression in

normal MLO-Y4 cells and MLO-Y4 cells treated with the PPARα antagonists (MK886 and GW6471), PPARβ/δ antagonists (GSK0660 and GSK3787), and PPARγ antagonists (T0070907 and GW9662). **(b)** Western blotting statistical analyses of the SERCA2 expression in HGHF-exposed MLO-Y4 cells treated with the PPARα agonists (Fenofibrate and GW7647), PPARβ/δ agonists (GW0742 and GW501516), and PPARγ agonists (rosiglitazone and pioglitazone). **(c-e)** Statistical analyses of intracellular Ca^{2+} signaling and gene and protein expression of osteocyte-related cytokines in HGHF-treated MLO-Y4 cells with lentiviral overexpression of PPARα in response to FSS stimulation. **(f-h)** Statistical analyses of intracellular Ca^{2+} signaling and gene and protein expression of osteocyte-related cytokines in MLO-Y4 cells with lentiviral silencing of PPARα in response to FSS stimulation. Graphs represent mean ± SD (**a, b, d, e, g, h:** *n*=6 biologically independent replicates; **c, f:** *n*=120 cells per group). **a-c, f** ***P<0.001 by one-way ANOVA with Bonferroni's post test. **d, e, g, h** *P<0.05, **P<0.01, and ***P<0.001 by two-way ANOVA with Bonferroni's post test. Specific *P* values are provided in the Source Data file.

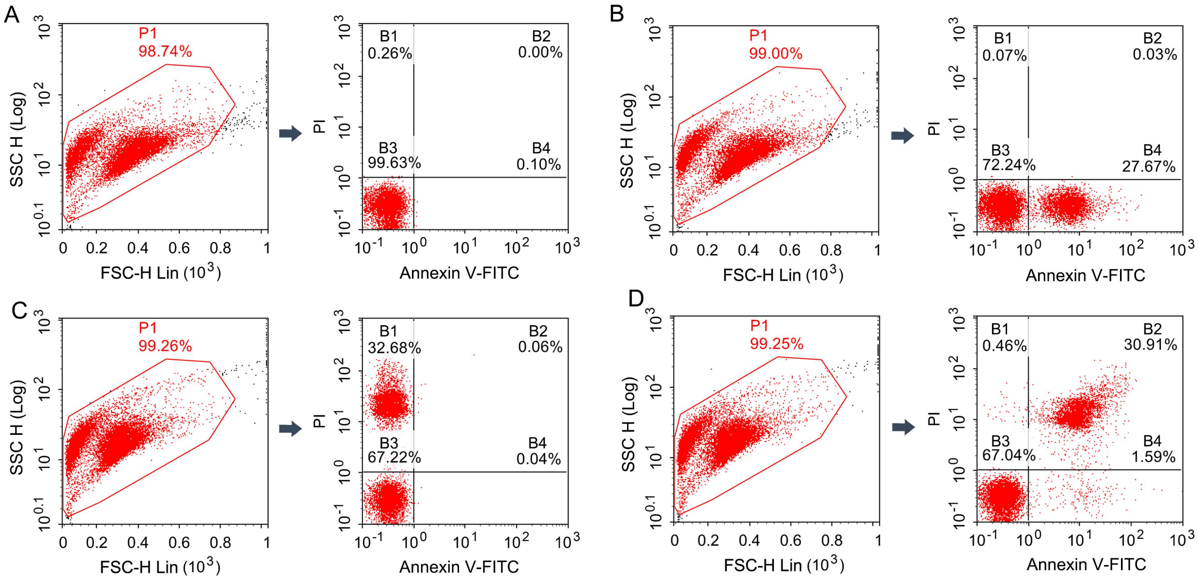

Fig. S19, related to Fig. 5. Gating stratgy for flow cytometry. (a) Quadrants were set based on the blank control group. **(b)** Compensation was set for the Annexin V-FITC channel. **(c)** Compensation was set for the propidium iodide (PI) channel. **(d)** Detecting the sample according to the above experimental conditions.

Table S1. Primers used for qRT-PCR and ChIP assays

Genes	Primers	Primer sequence (5'-3')
ATP2A1	Forward	GAACCGTGTCACAGATCCAGA
	Reverse	TTGGCTGAAGATGCATGGCT
ATP2A2	Forward	TTGTGGCCCGAAACTACCTG
	Reverse	GACCACCAGGGGCATAATGA
ATP2A3	Forward	GACGCTCACCACCAATCAGA
	Reverse	GCGCAGAATCATTGCAGAGG
β-catenin	Forward	CGCCGCTTATAAATCGCTCC
	Reverse	TTCACAGGACACGAGCTGAC
Opg	Forward	ACCAAAGTGAATGCCGAGAGAG
	Reverse	ACGCTGCTTTCACAGAGGTC
Tnfsf11	Forward	CACAGCGCTTCTCAGGAGCTC
	Reverse	GAGATCTTGGCCCAGCCTCGA
DKK1	Forward	GCTGCATGAGGCACGCTAT
	Reverse	GCGTTGTGGTCATTACCAAGGTT
Col1a1	Forward	GCTCCTCTTAGGGGCCACT
	Reverse	CCACGTCTCACCATTGGGG
Osx	Forward	ATGGCGTCCTCTCTGCTTGA
	Reverse	GAAGGGTGGGTAGTCATTTG
Runx2	Forward	GACTGTGGTTACCGTCATGGC
	Reverse	ACTTGGTTTTTCATAACAGCGGA
Nfatc1	Forward	GACTTCGATTTCCTCTTCGAGTTC
	Reverse	CTCGATTCTCGGACTCTCCAG
Calcr	Forward	AGTTGCCCTCTTATGAAGGAGAAG
	Reverse	GGAGTGTCGTCCCAGCACAT
Ctsk	Forward	ATATGTGGGCCAGGATGAAAGTT
	Reverse	TCGTTCCCCACAGGAATCTCT
TRAP	Forward	AAATCACTCTTTAAGACCAG
	Reverse	TTATTGAATAGCAGTGACAG
β-actin	Forward	CCAGGTCATCACTATTGGCAAGGA
	Reverse	GAGCAGTAATCTCCTTCTGCATCC

Table S2. Probes used for EMSA assays

Probes	Sequences (5'-3')
P1-WT	CATCAATAAGCTGGATTTATTTTGGCCTCTGGACCACTTACCTATGAG AAGAATGGC
P1-Mut	CATCAATAAGCTGGATTTATTTCCGCTGCGATTCGACTTACCTATGAG AAGAATGGC
P2-WT	GGGGCACTGAGAGTAGGAAGGTCATGACCTCTCTTAGTTTGTCTGTG ATGCTCACTG
P2-Mut	GGGGCACTGAGAGTAGGAAGGTCATCGCACTTCTTAGTTTGTCTGTG ATGCTCACTG

Table S3. RNA oligos

siRNA	Sense (5'-3')	Antisense (5'-3')
siSERCA2-#1	CUGCUAACUUCAUCAAAUATT	UAUUUGAUGAAGUUAGCAGTT
siSERCA2-#2	GUGUCAAACAGAAGAUUAUTT	AUAAUCUUCUGUUUGACACTT

shRNA	sequence (5'-3')
shPPARα-#1	CCCTTATCTGAAGAATTCTTA
shPPARα-#2	AGAAATTCTTACCTGTGAA
