# Supplementary material for: Rescuing SERCA2 pump deficiency improves bone mechano-responsiveness in type 2 diabetes by shaping osteocyte calcium dynamics
Source: Nat Commun. 2024 Jan 30;15:890. doi: 10.1038/s41467-024-45023-6 (PMC10828510; doi:10.1038/s41467-024-45023-6)
Supplement: Supplementary file 3 — Reporting Summary [file 41467_2024_45023_MOESM3_ESM.pdf]

nature portfolio

Corresponding author(s): Da Jing

Last updated by author(s): Jan 11, 2024

Reporting Summary

Nature Portfolio wishes to improve the reproducibility of the work that we publish. This form provides structure for consistency and transparency in reporting. For further information on Nature Portfolio policies, see our Editorial Policies and the Editorial Policy Checklist.

Statistics

For all statistical analyses, confirm that the following items are present in the figure legend, table legend, main text, or Methods section.

n/a	Confirmed	
☐	☒	The exact sample size (*n*) for each experimental group/condition, given as a discrete number and unit of measurement
☐	☒	A statement on whether measurements were taken from distinct samples or whether the same sample was measured repeatedly
☐	☒	The statistical test(s) used AND whether they are one- or two-sided<br>*Only common tests should be described solely by name; describe more complex techniques in the Methods section.*
☒	☐	A description of all covariates tested
☐	☒	A description of any assumptions or corrections, such as tests of normality and adjustment for multiple comparisons
☐	☒	A full description of the statistical parameters including central tendency (e.g. means) or other basic estimates (e.g. regression coefficient) AND variation (e.g. standard deviation) or associated estimates of uncertainty (e.g. confidence intervals)
☐	☒	For null hypothesis testing, the test statistic (e.g. *F*, *t*, *r*) with confidence intervals, effect sizes, degrees of freedom and *P* value noted<br>*Give P values as exact values whenever suitable.*
☒	☐	For Bayesian analysis, information on the choice of priors and Markov chain Monte Carlo settings
☒	☐	For hierarchical and complex designs, identification of the appropriate level for tests and full reporting of outcomes
☒	☐	Estimates of effect sizes (e.g. Cohen's *d*, Pearson's *r*), indicating how they were calculated

Our web collection on statistics for biologists contains articles on many of the points above.

Software and code

Policy information about availability of computer code

Data collection	Data were acquired by Confocal microscope (Fluoview FV3000, Olympus, Japan); iMark Microplate Reader (M680, Bio-Rad, CA); Fluorescence microscope (IX83, Olympus, Tokyo, Japan); Cytomics FC500 flow cytometer (Beckman Coulter, Miami, FL); Denovix DS-11 spectrophotometer (Denovix, Wilmington, USA); LightCycler 480 Real-time PCR System (Roche Applied Science, Penzberg, Germany); ECL system (Image Quant 350, GE Healthcare, UK); NovaSeq 6000 system (LC-Bio Technology CO., Ltd., Hangzhou, China); Micro-CT system (GE healthcare, Madison, WI); Nanoindenter (G200, Agilent Technologies, Chandler, AZ); mechanical testing machine (ElectroForce 3220, Bose Corp, Eden Prairie, MN, USA).
Data analysis	Data were analyzed by V31S-SW Fluoview software (Olympus, Japan); ImageJ 1.52a software (National Institutes of Health, USA) ; VGStudio Max 2.2 software (Volume Graphics, Heidelberg, Germany); Prism 9.5.1 software (GraphPad, USA); Origin 2021 software (OriginLab Corporation), Expo32 ADC Analysis (Beckman Coulter).

For manuscripts utilizing custom algorithms or software that are central to the research but not yet described in published literature, software must be made available to editors and reviewers. We strongly encourage code deposition in a community repository (e.g. GitHub). See the Nature Portfolio guidelines for submitting code & software for further information.

Data

Policy information about availability of data

All manuscripts must include a data availability statement. This statement should provide the following information, where applicable:

- Accession codes, unique identifiers, or web links for publicly available datasets
- A description of any restrictions on data availability
- For clinical datasets or third party data, please ensure that the statement adheres to our policy

The RNA-seq data have been deposited to SRA (Sequence read archive) under the accession number PRJNA901004. The genomic sequence of ATP2A2 was obtained from the Ensembl Genomes databases (http://asia.ensembl.org/index.html). The remaining data are available within the Article, and Supplementary Information. Source data are provided with this paper.

Research involving human participants, their data, or biological material

Policy information about studies with human participants or human data. See also policy information about sex, gender (identity/presentation), and sexual orientation and race, ethnicity and racism.

Reporting on sex and gender	N/A
Reporting on race, ethnicity, or other socially relevant groupings	N/A
Population characteristics	N/A
Recruitment	N/A
Ethics oversight	N/A

Note that full information on the approval of the study protocol must also be provided in the manuscript.

Field-specific reporting

Please select the one below that is the best fit for your research. If you are not sure, read the appropriate sections before making your selection.

☒ Life sciences ☐ Behavioural & social sciences ☐ Ecological, evolutionary & environmental sciences

For a reference copy of the document with all sections, see nature.com/documents/nr-reporting-summary-flat.pdf

Life sciences study design

All studies must disclose on these points even when the disclosure is negative.

Sample size	No statistical methods were used to predetermine sample sizes. We used generally accepted sample sizes in accordance to own previous experiences with reproducable differences between conditions indicating that the chosen sample sizes were sufficient. Our sample sizes are similar to those generally employed in the life sciences field.<br>All experiments were performed with at least 3 independent biological replicates per condition. At least 6 or 8 animals were included in each group for in vivo analyses and at least 6 samples were included in each group for in vitro studies. For calcium signaling anaysis, at least 120 cells were selected in each group. The exact numbers of samples are indicated on scatter dot plots in each figure and were elucidate in the correspongding legends.
Data exclusions	No data were excluded from the analysis.
Replication	Data were obtained in at least biologically independent replicates and the number of animals or experiments is described in corresponding figure legends.
Randomization	All C57BL/6J and KK-Ay mice and were randomly divided to the control and experimental groups. The cells were randomized into different groups before treatment. The transgenic mice (WT and SERCA2 cKI mice) were allocated based on results of PCR-genotyping performed 2 weeks after birth. After allocation, the SERCA2 cKI mice were randomized into different groups before HFD+STZ treatment.
Blinding	Data was blinded with respect to group allocation and data analysis of Ca2+ signaling analysis, cellular activity and bio-function analysis, animal histological and histomorphometrical assays, IHC staining and apoptosis and viability assays.

Reporting for specific materials, systems and methods

We require information from authors about some types of materials, experimental systems and methods used in many studies. Here, indicate whether each material, system or method listed is relevant to your study. If you are not sure if a list item applies to your research, read the appropriate section before selecting a response.

Materials & experimental systems

n/a	Involved in the study
☐	☒ Antibodies
☐	☒ Eukaryotic cell lines
☒	☐ Palaeontology and archaeology
☐	☒ Animals and other organisms
☒	☐ Clinical data
☒	☐ Dual use research of concern
☒	☐ Plants

Methods

n/a	Involved in the study
☒	☐ ChIP-seq
☐	☒ Flow cytometry
☒	☐ MRI-based neuroimaging

Antibodies

Antibodies used

Immunohistochemistry: Caspase-3 (1:200; Cell Signaling Technology, Cat# 9661, RRID: AB_2341188), RANKL (1:100; Proteintech, Cat# 66610-1-Ig, RRID: AB_2881970), OPG (1:100; Abcam, Cat# ab183910, RRID: AB_2934183), Sclerostin antibody (1:200; R&D systems, Cat# AF1589, RRID: AB_2195345), SERCA1 antibody (1:100; Affinity Biosciences, Cat# DF4449, RRID:AB_283604), SERCA2 antibody (1:100; Abcam, Cat# ab150435, RRID: AB_2910256), SERCA3 antibody (1:100; Proteintech, Cat# 13619-1-AP, RRID: AB_2061448) and PPARα (1:200; Affinity Biosciences, Cat# AF5301, RRID: AB_2837786) .

Western blotting: SERCA1 (1:500; Affinity Biosciences, Cat# DF4449, RRID:AB_283604), SERCA2 (1:5000; Abcam, Cat# ab150435, RRID: AB_2910256), SERCA3 (1:5000; Proteintech, Cat# 13619-1-AP, RRID: AB_2061448), IP3R (1:1000; Affinity Biosciences, Cat# AF6592, RRID: AB_2847316), PLCβ1 (1:1000; Proteintech, Cat# 26551-1-AP, RRID: AB_2861360), P2Y2 (1:1000; Affinity Biosciences, Cat# DF10259, RRID: AB_2840837), P2Y4 (1:1000; Affinity Biosciences, Cat# DF7163, RRID: AB_2839115), P2Y12 (1:2000; Abcam, Cat# ab184411, RRID: AB_2877713), Col1a1 (1:1000; Abcam, Cat# ab260043, RRID: AB_2922767), Runx2 (1:1000; Affinity Biosciences, Cat# AF5186, RRID: AB_2837672), Osx (1:1000; Bioss, Cat# bs-1110R, RRID: AB_10856257), β-catenin (1:1000; Proteintech, Cat# 51067-2-AP, RRID: AB_2086128), OPG (1:1000; Abcam, Cat# ab183910, RRID: AB_2934183), RANKL (1:1000; Proteintech, Cat# 66610-1-Ig, RRID: AB_2881970), DKK1 (1:2000; Proteintech, Cat# 21112-1-AP, RRID: AB_10733097), NFATc1 (1:500; Santa Cruz, Cat# sc-17834, RRID: AB_628011), Calcr (1:1000; Proteintech, Cat# 20868-1-AP, RRID: AB_2878754), Ctsk (1:1000; Affinity Biosciences, Cat# DF6614, RRID: AB_2838576), TRAP (1:2000; Abcam, Cat# ab52750, RRID: AB_868816), PPARα (1:1000; Affinity Biosciences, Cat# AF5301, RRID: AB_2837786), p-PPARα (1:1000; Affinity Biosciences, Cat# AF8392, RRID: AB_2840453) and β-actin (1:4000; Proteintech, Cat# 20536-1-AP, RRID: AB_10700003). HRP-conjugated goat-anti-rabbit secondary antibody (1:5000; Abcam, Cat# ab6721, RRID: AB_955447) and HRP-conjugated goat-anti-mouse secondary antibody (1:5000; Abcam, Cat# ab6789, Cat# ab6789).

Validation

All antibodies were purchased from commercial vendors and were validated by manufactures, other studies and/or in this study. We also provided associated datasheets link as below.

-SERCA1: Tested applications suitable for: WB 1:500-1:1000, IF/ICC 1:100-1:500, IHC 1:50-1:200. Species reactivity reacts with: Human, Mouse, Rat. (https://www.affbiotech.cn/goods-3581-DF4449-ATP2A1_Antibody.html)
-SERCA2: Tested applications suitable for: WB, IHC-P, ICC/IF, Flow Cyt (Intra). Species reactivity reacts with: Mouse, Rat, Human. (https://www.abcam.cn/products/primary-antibodies/serca2-atpase-antibody-epr9392-ab150435.html)
-SERCA3: Tested applications suitable for: IHC, IP, WB, ELISA. Species reactivity reacts with: Human, Mouse, Rat. (https://www.ptgcn.com/products/ATP2A3-Antibody-13619-1-AP.htm)
-IP3R: Tested applications suitable for: WB 1:500-1:2000. Species reactivity reacts with: Human,Mouse,Rat. (https://www.affbiotech.cn/goods-17678-AF6592-IP3_Receptor_Antibody.html)
-PLCβ1: Tested applications suitable for: IHC, WB,ELISA.Species reactivity reacts with: Human,Mouse,Rat. (https://www.ptgcn.com/products/PLCB1-Antibody-26551-1-AP.htm)
-P2Y2: Tested applications suitable for: IHC 1:50-1:200, WB 1:1000-3000, IF/ICC 1:100-1:500.Species reactivity reacts with: Human,Mouse,Rat. (https://www.affbiotech.cn/goods-11101-DF10259-P2RY2_Antibody.html)
-P2Y4: Tested applications suitable for: WB 1:500-1:2000, IHC 1:50-1:100, IF/ICC 1:100-1:500. Species reactivity reacts with: Human,Mouse,Rat. (https://www.affbiotech.cn/goods-5965-DF7163-P2RY4_Antibody.html)
-P2Y12: Tested applications suitable for: IP, WB. Species reactivity reacts with: Mouse, Rat. (https://www.abcam.cn/products/primary-antibodies/p2y12-antibody-epr18611-ab184411.html)
-Col1a1: Tested applications suitable for: IP, WB. Species reactivity reacts with: Mouse, Rat, Human. (https://www.abcam.cn/products/primary-antibodies/collagen-i-antibody-epr22894-89-ab260043.html)
-Runx2: Tested applications suitable for: WB 1:500-1:2000, IHC 1:50-1:200, IF/ICC 1:100-1:500. Species reactivity reacts with: Mouse, Rat, Human. (https://www.affbiotech.cn/goods-4493-AF5186-RUNX2_Antibody.html)
-Osx: Tested applications suitable for: WB=1:500-2000, ELISA=1:5000-10000, IHC-P=1:100-500, IHC-F=1:100-500, IF=1:100-500. Species reactivity reacts with: Mouse,Human. (http://www.bioss.com.cn/prolook_03.asp?id=AF08169606000660&pro37=1)
-β-catenin: Tested applications suitable for: FC, IF, IHC, IP, WB, ELISA. Species reactivity reacts with: Human, Mouse, Rat, Pig. (https://www.ptgcn.com/products/b-cat-Antibody-51067-2-AP.htm)
-OPG: Tested applications suitable for: WB, IHC-P. Species reactivity reacts with: Mouse, Human. (https://www.abcam.cn/products/primary-antibodies/osteoprotegerin-antibody-ab183910.html)
-RANKL: Tested applications suitable for: IF, WB, ELISA. Cited Applications: IF, IHC, WB. Species reactivity reacts with: Human, Mouse, Rat. (https://www.ptgcn.com/products/RANKL-Antibody-66610-1-Ig.htm#product-information)
-DKK1: Tested applications suitable for:IHC, WB, ELISA. Species reactivity reacts with: Human, Mouse, Rat. (https://www.ptgcn.com/products/DKK1-Antibody-21112-1-AP.htm)
-NFATc1: Tested applications suitable for:WB, IP, IF, IHC. Species reactivity reacts with: Human, Mouse, Rat. (https://www.scbt.com/

p/nfatc1-antibody-h-10?requestFrom=search)
-Calcr: Tested applications suitable for:WB, ELISA. Species reactivity reacts with: Human, Mouse. (https://www.ptgcn.com/products/CALCR-Antibody-20868-1-AP.htm)
-Ctsk: Tested applications suitable for:WB 1:500-1:2000, IHC 1:50-1:200, IF/ICC 1:100-1:500. Species reactivity reacts with: Human, Mouse, Rat. (https://www.affbiotech.cn/goods-5416-DF6614-CTSK_Antibody.html)
-TRAP: Tested applications suitable for:WB. Species reactivity reacts with: Human, Mouse. (https://www.abcam.cn/products/primary-antibodies/trapcd40l-antibody-ep462e-ab52750.html)
-PPARα: Tested applications suitable for:WB 1:500-1:2000, IHC 1:50-1:200, IF/ICC 1:100-1:500. Species reactivity reacts with: Human,Mouse,Rat. (https://www.affbiotech.cn/goods-4608-AF5301-PPAR_alpha_Antibody.html)
-p-PPARα: Tested applications suitable for:WB 1:1000-3000, IHC 1:50-1:200. Species reactivity reacts with: Human,Mouse,Rat,Monkey. (https://www.affbiotech.cn/goods-10715-AF8392-Phospho_PPAR_alpha_Ser12_Antibody.html)
-β-actin: Tested applications suitable for:WB FC, IF, IHC, WB, ELISA. Species reactivity reacts with: Human, Mouse, Rat. (https://www.ptgcn.com/products/ACTB-Antibody-20536-1-AP.htm)
-Goat Anti-Rabbit IgG H&L (HRP): Tested applications suitable for: IHC-P, WB, ELISA, Immunomicroscopy, Dot blot, ICC, IHC-Fr (https://www.abcam.cn/products/secondary-antibodies/goat-rabbit-igg-hl-hrp-ab6721.html)
-Goat Anti-Rabbit IgG H&L (HRP): Tested applications suitable for: ICC, IP, Dot blot, ELISA, IHC-P, IHC-Fr, Immunomicroscopy, WB (https://www.abcam.cn/products/secondary-antibodies/goat-mouse-igg-hl-hrp-ab6789.html)

Eukaryotic cell lines

Policy information about cell lines and Sex and Gender in Research

Cell line source(s)

Primary osteoblasts were isolated from male mouse calvaria. The RAW264.7 cells were purchased from ATCC. MLO-Y4 osteocytic cells were kindly gifted by Dr. Lynda Bonewald (University of Missouri-Kansas City, MO)

Authentication

Primary osteoblasts were authenticated based on the immunofluorescence staining and western blotting assays of Cbfα1. Raw264.7 cells were incubated with 50 ng/ml RANKL (Peprotech, Rocky Hill, NJ) to induce differentiation into osteoclast-like cells and were authenticated based on tartrate resistant acid phosphatase (TRAP) staining.
MLO-Y4 cells were authenticated upon the robust DMP-1 mRNA up-regulation following growth for >7 days at 37°C by qRT-PCR assays (DMP-1, Forward: 5'-CTGAAGAGAGGACGGGTGATT-3', Reverse: 5'-CGTGTGGTCACTATTTGCCTG-3'; GAPDH, Forward: 5'-TGTGTCCGTCGTGGATCTGA-3', Reverse: 5'-TTGCTGTTGAAGTCGCAGGAG-3').

Mycoplasma contamination

All cell lines were tested and confirmed to be mycoplasma negative.

Commonly misidentified lines
(See ICLAC register)

No commonly misidentified lines were used in this study.

Animals and other research organisms

Policy information about studies involving animals; ARRIVE guidelines recommended for reporting animal research, and Sex and Gender in Research

Laboratory animals

Four-week-old male C57BL/6J mice were purchased from the Animal Center of the Fourth Military Medical University, and were randomly assigned to the non-diabetes group and the experimentally-induced T2D group via the high-fat diet/streptozotocin treatment (HFD+STZ). Mice in the non-diabetes group were fed with a standard commercial chow diet (#DOSSYJY-001, DOSSY), and mice in the HFD+STZ group were fed with a high-fat diet with 60 kcal% fat (#D12492, Research Diets) throughout the experimental period. Eight-week-old male and female KK-Ay mice were purchased from Beijing Huafukang Bioscience Co. Ltd (Beijing, China) and fed with a high-fat diet throughout the experiment. C57BL/6 mice with SERCA2 gene specifically overexpression in osteocytes (SERCA2flox/flox; DMP1-Cre) were created based on the Cre/loxP system. DMP1-Cre transgenic mice (with the DMP1-Cre gene promoter drives Cre expression in osteocytes) were generated by GemPharmatech Co., Ltd (Nanjing, China). The floxed SERCA2 (SERCA2flox/flox) mice were generated by Cyagen Biosciences Inc. (Guangzhou, China). The SERCA2flox/flox mice were crossed with the DMP1-Cre mice to produce SERCA2flox/+; DMP1-Cre mice. The offspring were then intercrossed to generate SERCA2flox/flox; DMP1-Cre (termed SERCA2 cKI) mice. Mice were fed with a high-fat diet, and subjected to daily STZ (40 mg/kg) intraperitoneal injection for 5 consecutive days since 8 weeks of age. Mice were housed under a 12-h light/dark cycle at an ambient temperature of 23 ± 1°C and a relative humidity of 55 ± 5%.

Wild animals

No wild animals were used in the study.

Reporting on sex

Four-week-old male C57BL/6J mice, and eight-week-old male and female KK-Ay mice were used in this study.

Field-collected samples

No field-collected samples were used in the study.

Ethics oversight

Animal studies were approved and carried out according to the guidelines of the Institutional Animal Care and Use Committee of the Fourth Military Medical University (20190210), and in compliance with ARRIVE guidelines.

Note that full information on the approval of the study protocol must also be provided in the manuscript.

Plants

Seed stocks: N/A

Novel plant genotypes: N/A

Authentication: N/A

Flow Cytometry

Plots

Confirm that:

☒ The axis labels state the marker and fluorochrome used (e.g. CD4-FITC).

☒ The axis scales are clearly visible. Include numbers along axes only for bottom left plot of group (a 'group' is an analysis of identical markers).

☒ All plots are contour plots with outliers or pseudocolor plots.

☒ A numerical value for number of cells or percentage (with statistics) is provided.

Methodology

Sample preparation: The flow cytometry assays were performed for the apoptosis analysis of MLO-Y4 osteocytic-like cells by using an Annexin V-FITC apoptosis detection kit (Cat. No. KGA105-KGA108, KeyGEN, Jiangsu, China). Cells in different groups were digested with 0.25% trypsin (without EDTA) and washed with PBS for twice (2000rpm centrifugation for 5min each) and 1~5×10^5 cells were collected for analysis. Cells were resuspended in 500 µl binding buffer containing 5 µl Annexin V-FITC and 5 µl propidium iodide (PI), and incubated in the dark for 15 min. The degree of cell apoptosis was measured by a flow cytometry within 1 h.

Instrument: Beckman-Coulter XL flow cytometer (Beckman Coulter, Miami, FL)

Software: EXPO32 ADC software for the evaluation of cellular apoptosis of MLO-Y4 osteocytic-like cells

Cell population abundance: Up to 10,000 cells were selected in running apoptotic analysis for each biological duplicate. Representative apoptosis results shown in Figure 5 demonstrated abundance of distinct cell populations.

Gating strategy: Preliminary gates were set to exclude necrotic cells and debris on FSC/SSC plots. Then the left single cells were gated on the FSC-H/FSC-H plots in the log mode. Apoptotic cells positive for Annexin V-FITC can be seen in the bottom right quadrant and apoptotic cells positive for both Annexin V-FITC and PI in the top right quadrant. Cells negative for both stains (bottom left quadrant) were viable and not undergoing apoptosis.

☒ Tick this box to confirm that a figure exemplifying the gating strategy is provided in the Supplementary Information.
